# Promising AAV.U7snRNAs vectors targeting *DMPK* improve DM1 hallmarks in patient-derived cell lines

**DOI:** 10.3389/fcell.2023.1181040

**Published:** 2023-06-15

**Authors:** Camila F. Almeida, Florence Robriquet, Tatyana A. Vetter, Nianyuan Huang, Reid Neinast, Lumariz Hernandez-Rosario, Dhanarajan Rajakumar, W. David Arnold, Kim L. McBride, Kevin M. Flanigan, Robert B. Weiss, Nicolas Wein

**Affiliations:** ^1^ Center for Gene Therapy, Nationwide Children’s Hospital, Columbus, OH, United States; ^2^ Center for Cardiovascular Research, Nationwide Children’s Hospital, Columbus, OH, United States; ^3^ Department of Neurology, The Ohio State University, Columbus, OH, United States; ^4^ Department of Physical Medicine and Rehabilitation, University of Missouri School of Medicine, Columbia, MO, United States; ^5^ Department of Medical Genetics, University of Calgary, Calgary, AB, Canada; ^6^ Department of Pediatrics, The Ohio State University, Columbus, OH, United States; ^7^ Department of Human Genetics, The University of Utah School of Medicine, Salt Lake City, UT, United States

**Keywords:** aav, U7snRNA, myotonic dystrophy, gene therapy, spliceopathy

## Abstract

Myotonic dystrophy type 1 (DM1) is the most common form of muscular dystrophy in adults and affects mainly the skeletal muscle, heart, and brain. DM1 is caused by a CTG repeat expansion in the 3′UTR region of the *DMPK* gene that sequesters muscleblind-like proteins, blocking their splicing activity and forming nuclear RNA *foci*. Consequently, many genes have their splicing reversed to a fetal pattern. There is no treatment for DM1, but several approaches have been explored, including antisense oligonucleotides (ASOs) aiming to knock down *DMPK* expression or bind to the CTGs expansion. ASOs were shown to reduce RNA *foci* and restore the splicing pattern. However, ASOs have several limitations and although being safe treated DM1 patients did not demonstrate improvement in a human clinical trial. AAV-based gene therapies have the potential to overcome such limitations, providing longer and more stable expression of antisense sequences. In the present study, we designed different antisense sequences targeting exons 5 or 8 of *DMPK* and the CTG repeat tract aiming to knock down DMPK expression or promote steric hindrance, respectively. The antisense sequences were inserted in U7snRNAs, which were then vectorized in AAV8 particles. Patient-derived myoblasts treated with AAV8. U7snRNAs showed a significant reduction in the number of RNA *foci* and re-localization of muscle-blind protein. RNA-seq analysis revealed a global splicing correction in different patient-cell lines, without alteration in *DMPK* expression.

## Introduction

Myotonic dystrophy type 1 (DM1) is the most common form of muscular dystrophy among adults, with a prevalence of approximately 1 in 8,000 individuals in European populations [for review see (Thornton, 2014)]. The symptoms include myotonia, progressive muscle wasting, and weakness, abnormal cardiac conductions, gastrointestinal problems, endocrine dysfunctions, and cognitive impairment, among others, defining DM1 as a multisystem disorder. DM1 is caused by a CTG repeat expansion in the 3′ untranslated (UTR) region of the dystrophia myotonica protein kinase gene (*DMPK*) (Fu et al., 1992; Mahadevan et al., 1992) inherited with an autosomal dominant pattern. The number of CTG repeats correlates with the penetrance and severity of the associated disease, with healthy individuals having 5–37 repeats and pre-mutations with 38–49 repeats. DM1 cases with late adult onset are linked to a range of 50–150 CTG repeats, while those with young adult onset have a range of greater than 150–1,000 repeats, and CTG repeats surpassing 1,000 are associated with childhood and neonatal onset (Johnson et al., 2021).

At the mRNA level, the CUG repeats form secondary structures that aggregate in the nucleus and are referred to as RNA *foci* (Taneja et al., 1995; Davis et al., 1997). These nuclear aggregates also contain CUG-binding proteins, such as muscleblind-like proteins (MBNL), which are involved in mRNA processing, including alternative splicing (Wang et al., 2012). Consequently, the levels of free MBNL1 available are dramatically reduced. In opposition, the alternative splicing regulator CUG-binding protein 1 (CELF1) becomes hyperphosphorylated and has its activity enhanced (Kuyumcu-Martinez et al., 2007). MBNL1 and CELF1 have antagonistic activities and, together, control the alternative splicing of many genes. In DM1, since MBLN1 is trapped in RNA *foci*, CELF1 becomes more active and switches the alternative splicing pattern in favor of fetal-like isoforms (Sicot et al., 2011). The genes with altered splicing have been associated with specific DM1 symptoms. For example, the altered splicing of *BIN1*, *CLCN1*, *DMD*, *LDB3*, and *INSR*, have been related to muscle weakness, myotonia, myofiber disorganization, sudden cardiac death, and insulin resistance, respectively (Mankodi et al., 2002; Dansithong et al., 2005; Fugier et al., 2011; Rau et al., 2015; Yamamoto et al., 2019).

There is no effective treatment for DM1, but several pharmacological and genetic therapeutic strategies have been developed and tested in cellular and murine models of DM1. These therapies comprise a diverse range of strategies targeting the disease at different levels, from the DNA level with gene editing to modulation of downstream signaling pathways [recently reviewed in (Izzo et al., 2022)]. At the RNA level, different approaches specifically target the mutant mRNA and the interaction of MBNL1 with the CUG repeats. Modified antisense oligonucleotides (ASOs) were designed to interfere with the interaction of MBNL1 and the CUG repeats or to promote *DMPK* mRNA degradation. ASOs targeting the CUG repeats with different chemical modifications have been delivered to cell and animal models, like PMO-CAG25 (Wheeler et al., 2009), 2′-OMe-CAG (Mulders et al., 2009; González-Barriga et al., 2013), LNA-CAG (Nakamori et al., 2011), and all-LNA-CAG (Wojtkowiak-Szlachcic et al., 2015). Peptide-conjugated ASOs (PMO-CAG) have also been developed to enhance cell penetration (Leger et al., 2013; Klein et al., 2019). Gapmer ASOs were also designed to promote *DMPK* silencing by targeting regions upstream or downstream of the CTG repeats (Wheeler et al., 2012; Pandey et al., 2015; Jauvin et al., 2017; Yadava et al., 2020; Hu et al., 2021; Ait Benichou et al., 2022). Overall, in preclinical studies, these different classes of ASOs have shown to be successful in reducing RNA *foci* quantity, restoring the alternative splicing profile, and even abolishing myotonia in animal models. Based on encouraging preclinical data, a version of an MOE gapmer was administered to DM1 patients in a clinical trial, however, drug levels in skeletal muscles were not sufficient to reach therapeutical benefit and the trial was suspended. ASOs present a series of limitations that may prevent their successful application in humans. First, to sustain the therapeutic effect, they need to be repeatedly injected. Second, unless directly injected into the target tissue, they have restricted biodistribution. Third, to achieve therapeutic effects, high doses may be necessary.

To overcome the limitations and challenges of ASOs, we sought to develop adeno-associated virus-based (AAV) gene therapy strategies to treat DM1. Here, we report for the first time the use of AAV vectors to deliver U7 small nuclear RNA constructs targeting the coding region of *DMPK* or the 3′UTR region and CTG expansion tract in different patient-derived cell lines. The most efficient vectors were able to significantly reduce the number of nuclear RNA *foci* and change MBNL1 localization. Consequently, through RNA-seq analysis, we identified a global alternative splicing profile change, including in genes already known to be affected in DM1 and important for muscle function. Altogether, our data show that AAVs can be a reliable vector to deliver U7snRNAs that can efficiently rescue the spliceopathy characteristic of DM1.

## Methods

### Primary fibroblasts and cell transduction

Following informed consent obtained under an Institutional Review Board-approved protocol (IRB #00002860, IRB17-00719, and IBS00000123), a skin punch biopsy was performed under local anesthesia. The patient genotypes were obtained from their medical records. CTG lengths were analyzed for diagnosis in blood samples by Southern blot. Human fibroblasts were derived from skin biopsies of healthy individuals and DM1 patients. The biopsies were cut into small pieces (about 2 mm^2^) and each piece was placed in a 6-well plate and kept until it started to dry for 15 min, and 2 mL of growth medium composed of DMEM (Thermo Scientific, 10,569), 20% fetal bovine serum (Thermos Scientific, 16,000), 1% Antibiotic-Antimycotic (Thermo Scientific, 15,240) was carefully added to the well. The medium was changed after the first fibroblasts were attached, and then every two to 3 days until confluence was reached. After splitting cells, some unmodified primary cells were frozen. For viral transduction, cells were plated in a 12-well plate. At 50% confluency, 2 to 5 × 10^9^ vg/mL of each lentivirus (hTERT-puromycin and doxycycline-inducible MyoD-hygromycin) were added to 400 µL of growth medium. The following day, 1 mL of growth media was added. One or 2 days later, the cells were split in a 6-well plate and grew until reaching 70% confluence. At this step, the growth medium was supplemented with 400 μg/mL of hygromycin and 1 μg/mL of puromycin to select cells that integrate both lentiviruses. The cells were selected for at least 12 days, with medium refreshed every two to 3 days, and then named FibroMyoD (FM).

### Fibroblast conversion and myogenic differentiation

FibroMyoD (FM) cells were grown in growth media until cells reached 80% confluency. At that point, growth media was changed to Skeletal Muscle Cell Growth medium (Promocell, C23060), supplemented with 5 uM CHIR 99021 (Axon Medchem, 1,386), 10 uM DAPT (Tocris, 2634), 8 ug/mL doxycycline (Fisher Scientific, BP2653-5), which induces the expression of *MYOD* and consequent conversion of fibroblasts into myoblasts. After three to 4 days, the cells acquired an elongated and fusiform morphology and became oriented parallel to each other. At this point, cells were washed with PBS, and the myoblast growth medium was replaced by Skeletal Muscle Cell differentiation medium (Promocell, C23061), supplemented with CHIR 99021, DAPT, and doxycycline, to induce the fusion and differentiation of myoblasts into multinucleated myotubes. The medium was changed every other day for 7 days when cells were fixed with 4% PFA for immunofluorescence staining.

### Constructs

We designed three different antisense sequences targeting the splicing sites of *DMPK* exon 5 and three for exon 8 to induce the skipping of such exons (Figure 2A). We used Human Splicing Finder tool to identify splicing motifs important for exon inclusion. Then, we designed sequences to mask them and exclude the targeted exon from the final mRNA. Additional antisense sequences were designed to target the 3′UTR region. The 15CTG and 20CTG sequences are simple concatenated CTG trinucleotides. For the 5′ and 3′CTG sequences, we designed sequences targeting the CTG repeat expansion but also to be more specific to the DMPK gene, we expanded our sequence to either the 5′ or 3′ UTR of the DMPK gene as different regions of the human genome contain repeated CTG sequences. The antisense sequences were inserted in the mouse U7snRNA gene, which was cloned in a self-complementary AAV vector. The final constructs were verified by sequencing. The integrity of the inverted terminal repeats in the AAV backbone was verified by SmaI digestion.

### AAV cells transduction treatment

scAAV8. U7snRNAs particles were produced by Astellas Pharma Inc. (former Audentes). We selected three cell lines containing the largest numbers of repeats: 900, 1,150, and 1,450 CTGs. 7.5E+5 FM cells were plated on 10 cm Petri dishes and 5.5E + 4 FM cells were seeded on glass chamber slides. Once the cells reached 80% confluence, they were transduced with AAVs diluted in Skeletal Muscle Cell Growth medium (Promocell, C23060), supplemented with 5 uM CHIR 99021 (Axon Medchem, 1,386), 10 uM DAPT (Tocris, 2634), 8 ug/mL doxycycline (Fisher Scientific, BP2653-5). A total of 9E + 12vg or 6E + 11vg AAV particles were added to the Petri dishes and glass chamber slides, respectively. The same dose was used for all different AAVs and cells. However, due to limited AAV8. U7snRNA.20CTG volume, 1150CTG FM cells on the Petri dish received approximately half of the AAV amount. Two days after transduction, the growth medium was replaced by Skeletal Muscle Cell differentiation medium (Promocell, C23061), supplemented with CHIR 99021, DAPT, and doxycycline. Medium was refreshed every other day for 7 days when the cells on the glass slides were fixed on 4% PFA and cells on Petri dishes were pelleted and frozen at −80°C. Experimental design is shown in Figure 2B.

### RNA fluorescence *in situ* hybridization (RNA FISH)

FM cells were grown on glass chamber slides (Thermo Scientific Lab-TEK II CC2, 154,852), coated with Matrigel (Corning, 354,230) diluted 1:10, and fixed with 4% paraformaldehyde for 10 min. After fixation, cells were rinsed three times with 1X PBS, permeabilized with 0.1% TritonX-100 for 5 min, followed by a wash in 30% formamide, 2X SSC (saline sodium citrate buffer) for 10 min. Next, cells were hybridized with 2 ug/mL BSA, 66 μg/mL yeast tRNA, 1 ng/µL Cy3-labeled-(CAG)10 probe in 30% formamide, 2X SSC, for 3 h at 37°C. After hybridization, cells were washed in 30% formamide, 2X SSC for 30 min, at 37°C, and then in 1X SSC for 30 min, at room temperature under agitation. Next, cells were washed three times with 1X PBS. For MBNL1 co-staining, cells were incubated for 1 h in a blocking solution (5% normal goat serum, 0.3% Triton X-100 in PBS). Primary MBNL1 antibody (Abcam, ab45889) was diluted 1:250 in 1% BSA, 0.3% Triton X-100 in PBS solution and incubated overnight at 4°C. Cells were then washed three times with 1X PBS and incubated for 1 h with secondary antibody goat anti-rabbit Alexa Fluor 488 (Thermo Fisher, A11008) diluted 1:1,000 in 1% BSA, 0.3% Triton X-100 solution overnight at room temperature. After secondary incubation, the cells were rinsed three times with 1X PBS, and nuclei were stained with NucBlue™ Live ReadyProbes™ (Thermo Scientific, R37605) in 1X PBS for 20 min, at room temperature. Then, cells were washed three times with 1X PBS, and the coverslips were mounted on slides with Vectashield (Vector Laboratories, H-1000). The slides were imaged in a Nikon Ti2 Eclipse microscope, with a ×40 objective. Five large scan images were acquired of each cell line/treatment, comprising at least 2,000 nuclei per image. Quantification of *foci* and MBNL1 quantification was done in Nikon Elements software, using customized scripts.

### Immunofluorescence

After fixation, mature myotubes were permeabilized with 0.5% TritonX-100 for 10 min under agitation. Blocking was done in PBS with 10% goat serum (Thermo Scientific, 10,000 C) for 10 min under agitation. Primary antibody against myosin heavy chain (Developmental Studies Hybridoma Bank, MF-20 supernatant) was diluted 1:50 in blocking buffer, and cells were incubated for 2 hours at room temperature followed by incubation with secondary antibody goat anti-mouse Alexa Fluor 488 (Thermo Scientific, A-11001), diluted 1:500 in blocking buffer, for 1 hour at room temperature. Nuclei were counterstained with DAPI and slides were mounted with Vectashield. Images were captured in the Nikon Ti2 Eclipse microscope, with a ×20 objective, and processed in Nikon Elements software.

### RNA isolation and sequencing

Total RNA was extracted with TRIzol reagent (Fisher BioReagents, BP2805-25), followed by concentration and purification with Zymo kit, including DNase treatment to remove genomic DNA. RNA integrity was assessed with RNA 6000 Nano kit (Agilent, 5067-1511) in the 2100 Bioanalyzer. rRNA was depleted with NEB Next rRNA Depletion Kit (human, mouse, rat) (E #E6310X). For RNA-seq (mRNA) libraries generation, 200 ng of total RNA was treated with NEBNext rRNA Depletion kit (New England Biolabs, E6310X) and fragmented for 10 min. NEBNext Ultra II Directional (stranded) RNA Library Prep (New England Biolabs, E7760L) and NEBNext Multiplex Oligos (New England Biolabs, E6442L) kits were used for cDNA synthesis and amplification for 11 PCR cycles. The concentration of cDNA libraries was determined on Qubit Fluorometer (Thermo Fisher). Libraries were sequenced on an Illumina Novaseq SP Paired-End 100 bp format system.

### RNA-seq alignment and differential expression

The effective read length of the paired-end libraries was extended using the FLASH tool (Magoč and Salzberg, 2011) that merged overlapping paired-end reads. Extended read-pairs and non-extended forward reads were mapped using STAR v2.7.8c (Dobin et al., 2013) to the GRCh37/hg19 reference genome with splice junction annotations built using the GENCODE comprehensive gene annotation GTF file (gencode.v37lift37. annotation.gtf). STAR alignment options included: --twopassMode Basic --alignSJDBoverhangMin 1 --alignSJoverhangMin 8 --outFilterMismatchNoverLmax 0.3 --outFilterMultimapNmax 10. BAM alignments files were filtered for secondary alignments using the samtools view -h -F 2304 flag. Gene-level read counts were extracted from STAR output BAM files using featureCounts v.1.6.5 (Liao et al., 2014) with parameters set to strand-specific read counting. Differential gene expression analysis was performed using the edgeR Bioconductor package with the *voom* method (Smyth et al., 2018).

### Local splicing variation (LSV) detection

The MAJIQ/Voila v2.4.dev3 + g85d0781 software (Modeling Alternative Junction Inclusion Quantification) was used to detect and quantify splice variation from the filtered BAM alignment files (Vaquero-Garcia et al., 2016). MAJIQ Builder used Ensembl transcriptome annotations downloaded from http://majiq.biociphers.org/download/DB/ensembl.hg19.gff3. The 9 RNA-seq samples were configured as three experiments (NT, 3′CTG, and 20CTG) to construct splice graphs and detect known/novel local splicing variations. Relative inclusion values (“percent selected index” or PSI) of splice junctions within LSVs were calculated using the MAJIQ psi command with parameters minreads = 2 and minpos = 1. Pairwise delta PSI values (dPSI) were calculated for pairwise groups using the MAJIQ psi command with default parameters. The Voila view and modulize command used the --changing-between-group-dpsi 0.1 option.

### Gene ontology analysis

Gene Ontology analysis was carried out in WebGestalt online tool (Liao et al., 2019) to identify the enriched functional terms in “biological processes,” “cellular component,” and “molecular function” categories, using Over-Representation analysis (ORA), and FDR <0.05 and 6 as a minimum number of genes as threshold cutoffs. Weighted set cover post-processing was used to summarize and reduce redundancy and then identify the most representative GO terms.

### Reverse transcription, PCR amplification and quantification

Reverse transcription was performed with 1 µg of RNA with the RevertAid kit (Thermo Scientific, K1691) in a total volume of 20 µl. PCR amplification was performed using ×2 Master Mix (Thermo Scientific, K0172) and 150 ng of cDNA as template to analyze the splicing profile of selected genes (primer sequences are listed in Supplemental Table S1). PCR products were migrated on 2% agarose gel and stained with ethidium bromide. The band’s intensities were quantified on ImageLab software (BioRad), and the exon inclusion/exclusion was calculated as a ratio of the total intensities of the isoforms.

### Statistical analysis

Statistical analyses were performed in GraphPad Prism 9 software. Differences between multiple groups were assessed with Kruskal-Wallis test, followed by Dun’s multiple comparisons; *p* < 0.05 was considered significant.

## Results

### Fibroblast reprogramming and myoblast conversion and differentiation

**FIGURE 1:**
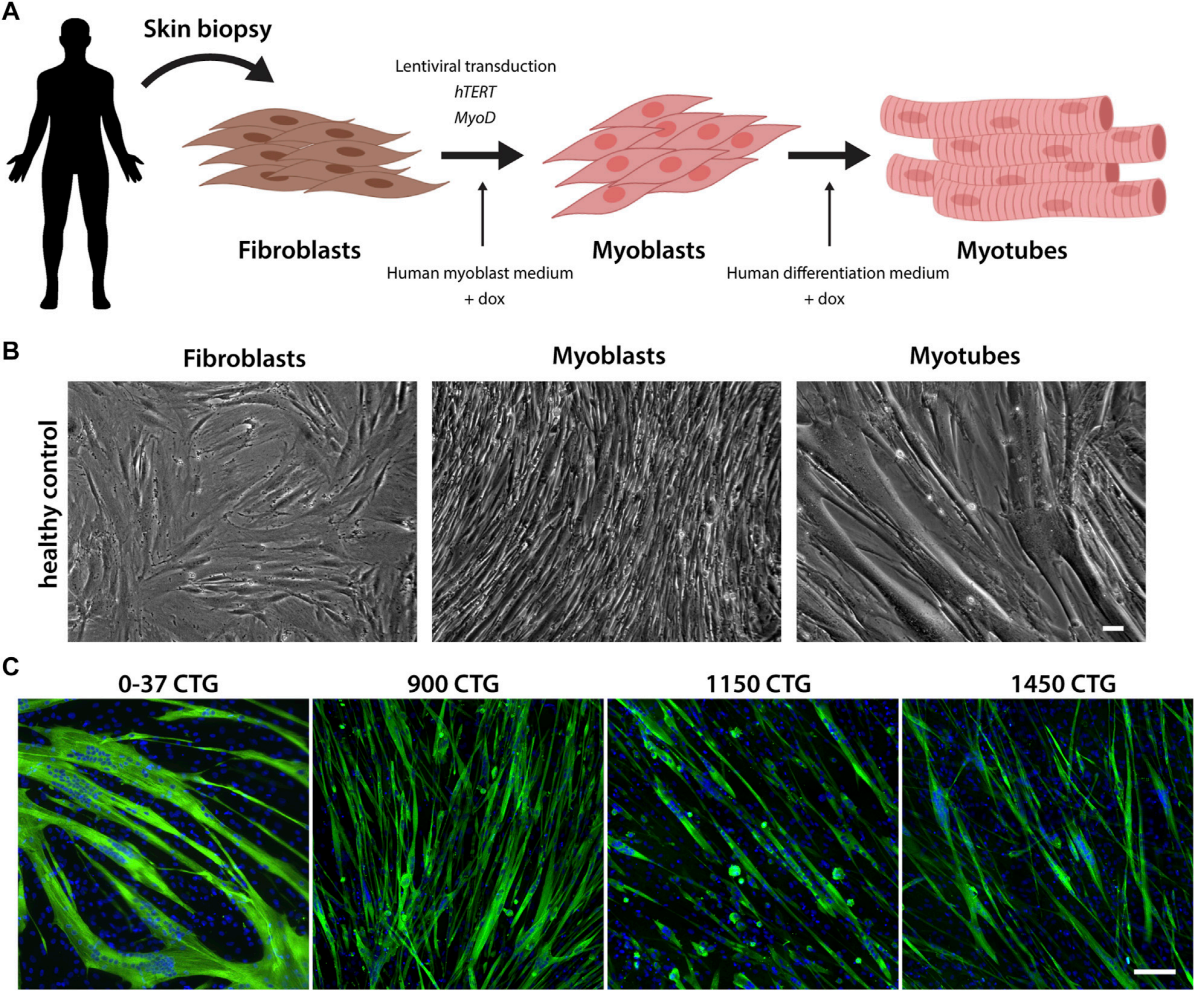
Reprogramming of fibroblasts into myogenic cells. **(A)**. Schematic representation of the fibroblast conversion into myotubes. Primary fibroblasts are derived from skin biopsies and co-transfected with lentiviruses carrying the hTERT and MYOD genes. Upon the addition of doxycycline, the transcription of MYOD is activated, inducing the activation of the myogenic program. Within 2–4 days, the fibroblasts acquire a myoblast morphology, and when differentiation medium is added, they start to fuse and form mature multinucleated myotubes. **(B)** Representative brightfield images of the conversion stages. Scale bar = 100 µm. **(C)** Differentiation of FM lines was confirmed by myosin heavy chain expression (green). Nuclei in blue. Scale bar = 100 µm.

To establish an *in vitro* cell model for DM1, we obtained primary fibroblasts derived from skin biopsies of patients with a broad range of mutations (Supplemental Table S2). The fibroblasts were transduced with two lentiviruses carrying the human telomerase (h*TERT*) and inducible-*Myod* genes. Since both lentiviruses contain selectable markers, co-transduced cells were submitted to antibiotic selection. After selection, the resulting cells were named FibroMyoD (FM). To induce the trans-differentiation, FM cells were cultured in myoblast growth medium, supplemented with doxycycline that activates the expression of *MYOD*. After two to 4 days, the fibroblasts became myoblasts, recognized by their elongated and fusiform morphology. Myoblasts were then induced to differentiate into multinucleated myotubes. A resumed scheme of the conversion process and representative images are shown in Figures 1A, B. The myogenic differentiation of all cell lines was evaluated by immunostaining of myosin heavy chain protein, expressed by mature myotubes but absent in undifferentiated fibroblasts and myoblasts (Figure 1C; Supplementary Figures S1A, B). Overall, the mutant cell lines presented a good differentiation as compared to healthy control cells, except for the lines harboring more than 900 CTG repeats that formed shorter myotubes containing fewer nuclei compared to the other lines (Figure 1C).

### RNA *foci* accumulation is significantly reduced after AAV8.U7snRNA transduction

DM1 pathology is believed to be caused by an RNA toxicity mechanism, in which the CUG repeats in *DMPK* mRNA form secondary structures that sequester proteins with affinity for CUG motifs, like MBNL1. MBNL1 protein is involved in mRNA processing, including alternative splicing of a wide variety of genes. Thus, MBNL1 sequestration in the RNA *foci* leads to a reduction in the availability of the protein that will ultimately alter the splicing profile of multiple genes, causing the manifestation of multisystemic symptoms in individuals with DM1. To revert RNA toxicity, it is necessary to reduce the number of RNA *foci* and that can be achieved by two general strategies. The first is the reduction of the number of expanded alleles through *DMPK* mRNA degradation. The second is to block the interaction of MBNL1 with the CUG repeats. Both strategies aim to restore the normal titration of MBNL1 and then rescue the abnormal splicing profile.

**FIGURE 2:**
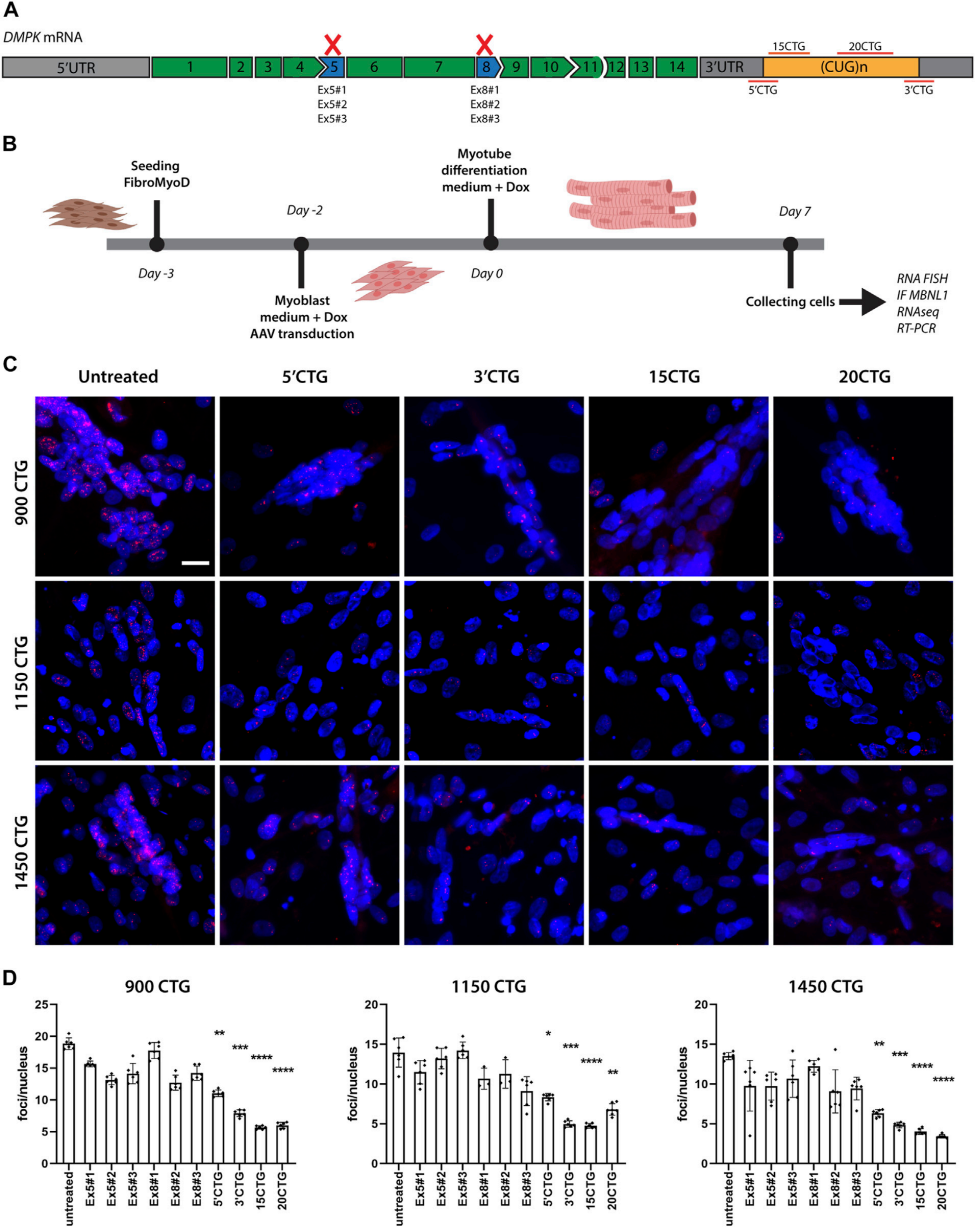
Delivery of U7snRNAs containing antisense sequences targeting the 3'UTR/CUG tract region decreases the number of RNA *foci*. **(A)** Schematic representation of antisense sequence targets. **(B)** Experimental design. **(C)** RNA FISH with Cy3-(CAG)_10_ probes in untreated and treated DM1 cell lines. RNA *foci* are recognized as discrete bright dots (in red) in nuclei. Nuclei counterstained with Hoechst 33342 (blue). Scale bar = 20 μm. **(D)** Quantification of *foci* number per nucleus before and after treated with the different constructs. Over 10,000 nuclei were quantified in each condition. Data are presented as mean with standard deviation. Kruskal-Wallis test followed by Dun's multiple comparisons to untreated samples. *p < 0.05; **p < 0.01; ***p < 0.001; ****p < 0.0001.

To knockdown *DMPK* mRNA expression, we designed antisense sequences to bind the splicing sites of exons 5 (named Ex5#1, Ex5#2, Ex5#3) and 8 (named Ex8#1, Ex8#2, Ex8#3) and then promote the skipping of these exons, causing the production of an out-of-frame transcript that is degraded by RNA decay mechanisms (Figure 2A). The antisense sequences were added to the mU7 gene and vectorized in AAV8 particles. After transducing patient-derived DM1 FM cells with AAV8, we performed RNA FISH to label and quantify the number of RNA *foci* per nucleus. Healthy cells were included as controls to confirm the absence of RNA *foci* when the cell has normal CTG repeats (Supplementary Figures S2A, B). These constructs did not promote a significant reduction in the number of *foci*/nuclei (Figure 2D), and neither rescued the splicing of genes assessed by RT-PCR (Figure 7) when compared to untreated cells. This can be attributed to a low skipping efficiency (Supplementary Figure S3A).

To promote steric hindrance of the CUG repeats, we designed four different antisense sequences targeting the *DMPK* mRNA 3′UTR region (Figure 2A). Two antisense sequences target the boundary of the 3′UTR and the CUG repeat tract at 5′ and 3′ of it (named 5′CTG and 3′CTG), conferring more specificity to the targets. Two antisense sequences consist of 15 and 20 CTG repeats (named 15CTG and 20CTG), that can bind multiple times along the length of the CUG repeats. These four sequences were also added to the mU7 gene and vectorized in AAV8. After only 7 days post-AAV8 treatment, all four constructs promoted a significant reduction in the number of *foci* per nucleus (Figure 2C; Supplementary Figure S4) as confirmed by *foci* quantification (Figure 2D) in three DM1 cell lines.

### MBNL1 is released from *foci* and re-localized in the nucleus

**FIGURE 3:**
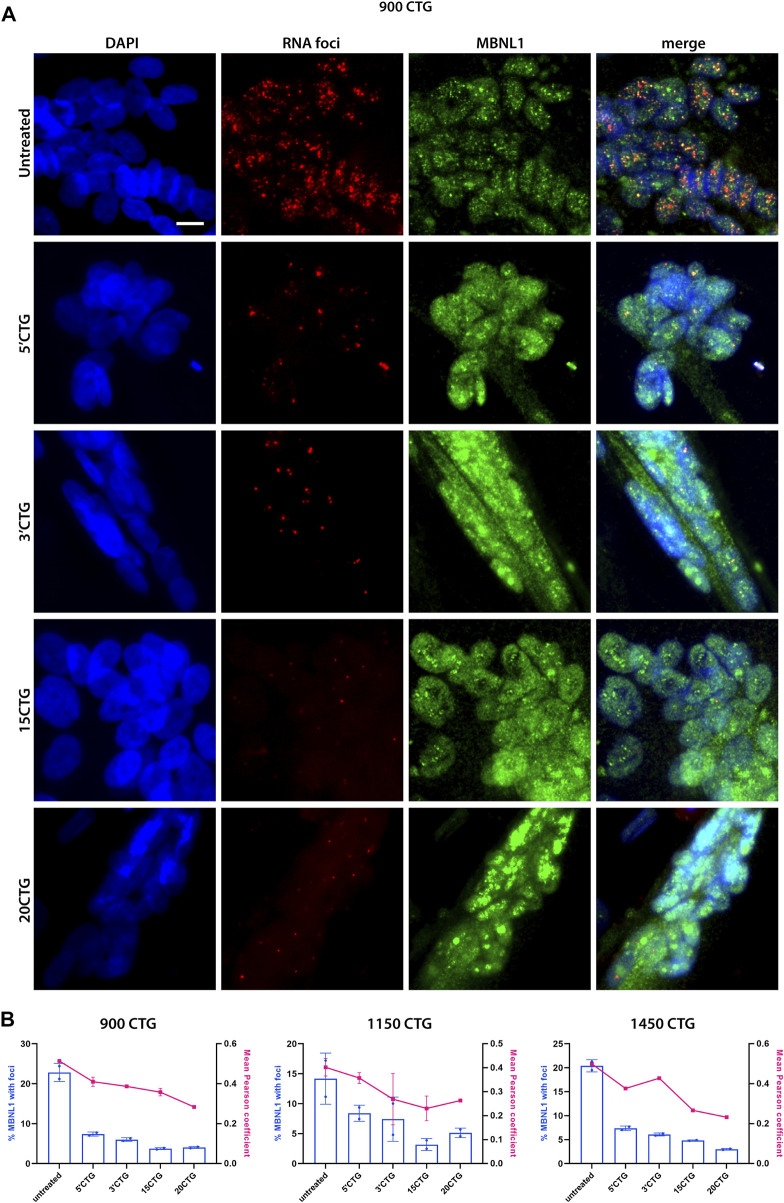
MBNL1 is released from RNA *foci* and re-localizes in the nucleoplasm. **(A)** RNA FISH (red) combined with immunofluorescence staining for MBNL1 (green) on 900CTG DM1 cell line, before and after treatment with AAV8.U7snRNAs targeting the 3’UTR/CUG tract region. In untreated cells, MBNL1 staining shows a dotted pattern and co-localizes with RNA *foci*, as seen in the merged image. After treatment, the number of RNA *foci* reduces dramatically, releasing MBNL1 which now shows a diffuse localization in the nucleus. Scale bar = 20 µm. **(B)** MBNL1 signal quantitative analysis. The bars represent the percentage of MBNL1 that co-localizes with foci and the line indicates the mean Pearson coefficient of correlation.

To determine whether the RNA *foci* reduction had an impact on MBNL1 localization, we did a co-staining of RNA *foci* by FISH and MBNL1 by immunofluorescence. Before treatment, we found MBNL1 co-localizing with RNA *foci* in the nuclei of differentiated myotubes of three DM1 cell lines carrying large numbers of repeats (Figure 3A; Supplementary Figures S5, 6). After treatment with the constructs targeting the 3′UTR/CUG repeats region, we saw a significant reduction in the number of *foci* per nucleus, as discussed above, accompanied by the release and redistribution of MBNL1 protein, as seen by the expected diffuse localization in the nucleus, especially in the cells treated with 3′CTG, 15CTG, and 20CTG constructs (Figure 3A; Supplementary Figures S2C, 5, 6). Quantitative analysis of the co-localization of MBNL1 with RNA *foci* confirmed the visual observations, showing decreased co-localization (Figure 3B). Pearson coefficient of correlation was calculated to show the relationship between RNA *foci* and MBNL1 signals. This coefficient ranges from −1 to 1, where values closer to 1 indicate a stronger correlation or more co-localization of MBN1 in RNA *foci* in this case. After treatment, a reduction in these values was observed (Figure 3B), confirming that the U7snRNAs can free MBNL1 from the *DMPK* expanded CUG repeats.

### Improved myoblast fusion

To assess myoblast fusion, we counted “distinct nuclear domains” based on DAPI staining area. A domain definition includes both isolated nuclei and nuclear clusters, therefore smaller number of distinct domains and larger size are indicative of more fusion because more differentiated myotubes tend to have more clustered nuclei. Our analysis showed that treated cells have a smaller number of nuclear domains, which we interpreted as an improved fusion index compared to untreated cells (Supplementary Figure S3B).

### Transcriptome assessment of differential gene expression

**FIGURE 4:**
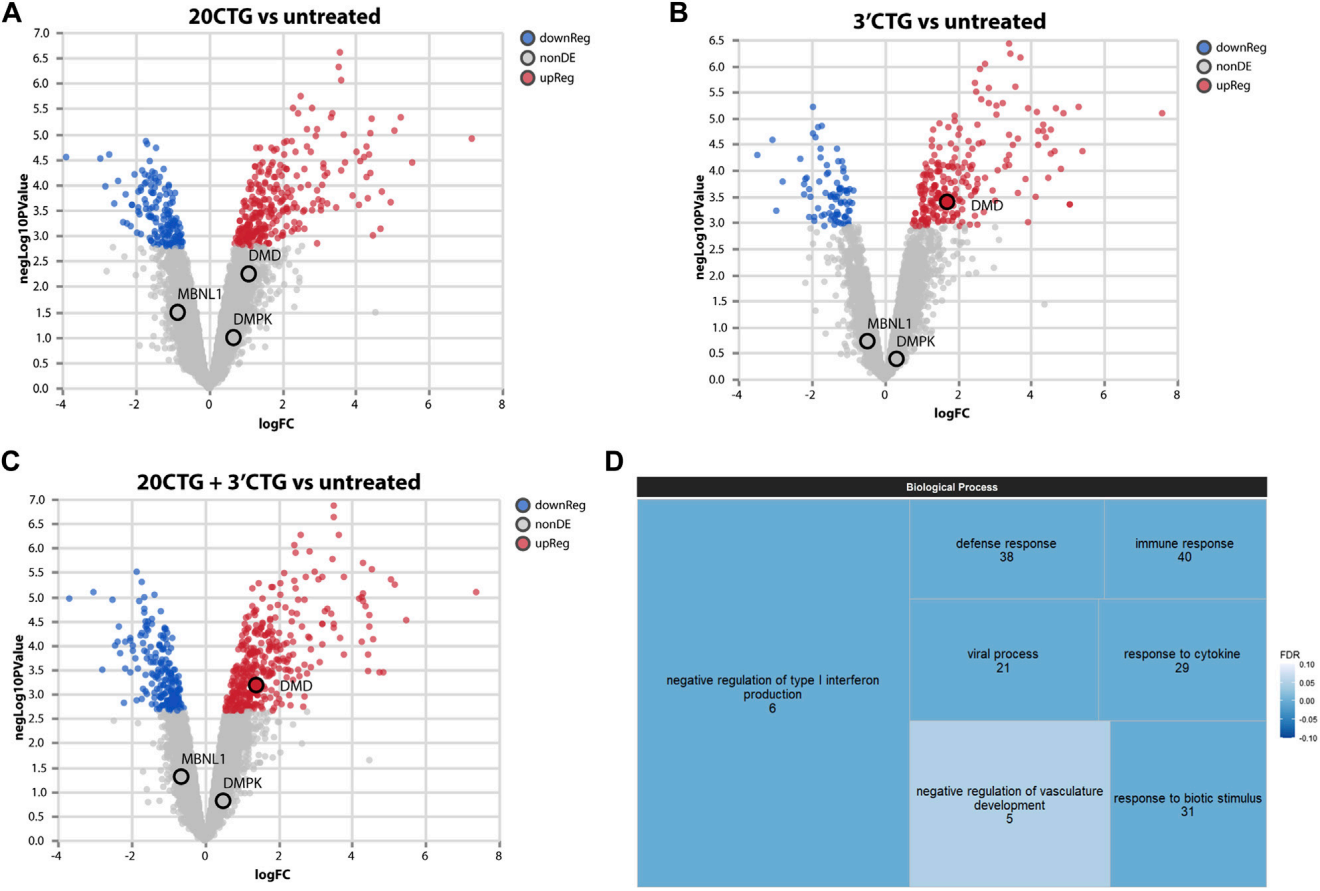
Transcriptome analyses of differentially expressed genes. Volcano plots showing up and downregulated genes in DM1 myotubes treated with 20CTG **(A)** and 3′CTG constructs **(B)**. **(C)** Volcano plot of the combined transcriptomes of 20CTG and 3′CTG treated myotubes in comparison to untreated cells, highlighting *DMD* transcript that is upregulated, and *MBNL1* and *DMPK* transcripts that are unchanged. **(D)** Tree map representing the Gene Ontology analysis of DEGs from C comparison. The size of the rectangles is proportional to the enrichment of the GO term. The numerals correspond to the number of DEGs from the input list involved in the GO term. The color gradient corresponds to the false discovery rate (FDR) where darker blue represents smaller FDR values. FDR cutoff was <0.05.

Next, we sought to investigate the global impact of the 3′CTG and 20CTG constructs on the transcriptome of the treated myotubes by RNA sequencing. We compared the transcriptome of treated myotubes versus untreated myotubes to identify differentially expressed genes (DEGs). DEG analysis assesses the gene expression of all transcripts of a given gene independently of alternative splicing. Only genes with fold-change values equal or greater/less than +2.00/−2.00 were considered differentially up- or downregulated, respectively (Supplemental Table S3). The myotubes treated with the 3′CTG and 20CTG constructs showed 112 and 120 DEGs, respectively (Figures 4A, B). We also combined the transcriptome of both conditions and compared it to untreated myotubes, and we found 111 DEGs (Figure 4C).

To understand the significance of these DEGs, we performed a Gene Ontology analysis to identify enriched biological processes in which these DEGs are involved. The most enriched and significant GO terms found are overall related to immune response (Figure 4D). The differential expression of these genes was not unexpected as a DM1 transcriptional signature, but it is probably a response to the AAVs. Since the cells were collected only 7 days after transduction, the immune mechanisms were still activated, but probably starting to decrease, as evidenced by the high enrichment of the GO term “negative regulation of type I interferon production”.

Interestingly, we found that the 3′CTG treatment upregulated *DMD* expression, while *MBNL1* and *DMPK* transcripts were unchanged (also in the 20CTG treatment). This suggests that RNA *foci* reduction is not accompanied by *DMPK* mRNA degradation and that the MBNL1 protein seen in the nucleus is no longer able to access and bind the CUG repeats.

### Correction of alternative splicing landscape

**FIGURE 5:**
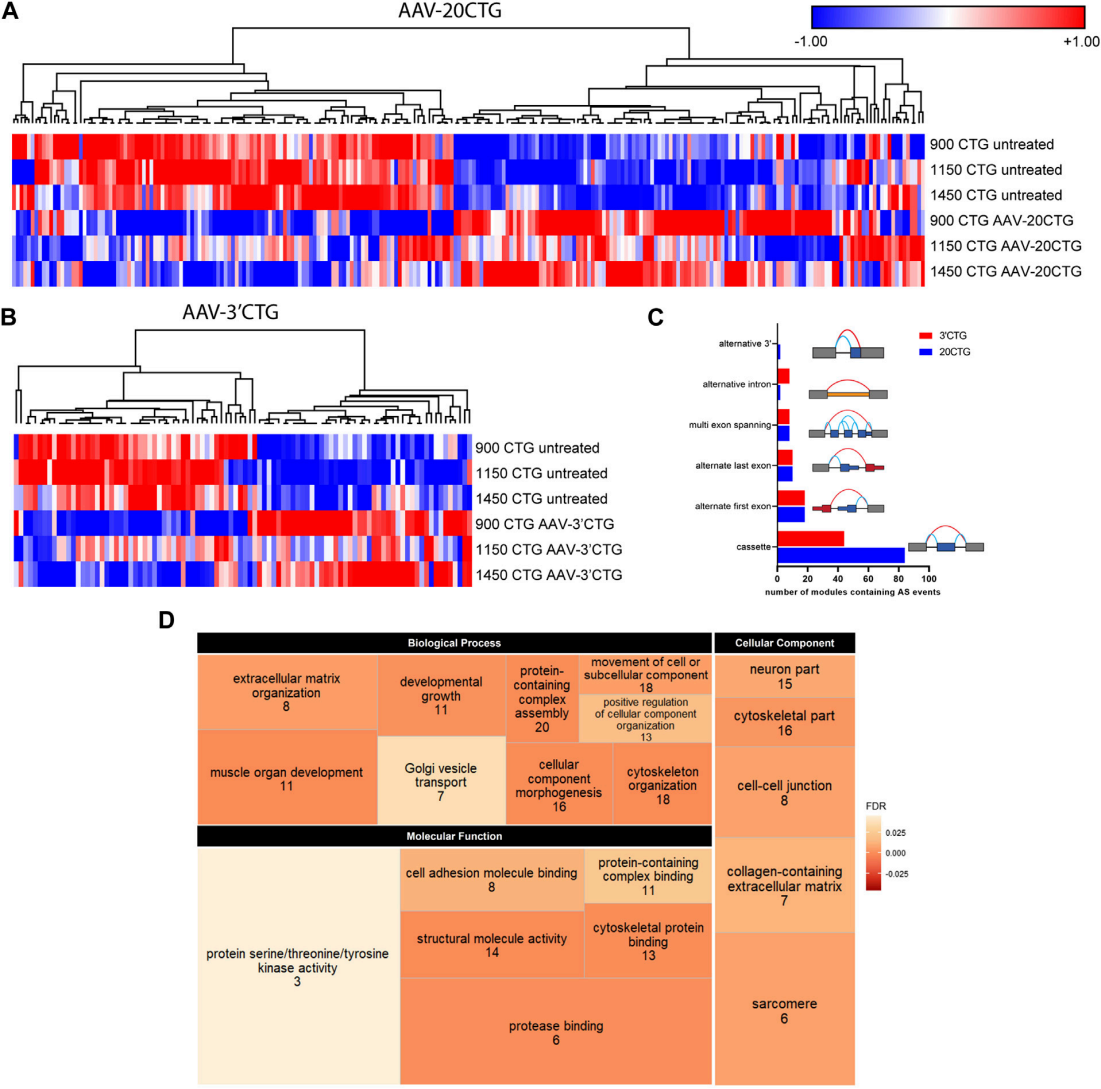
Alternative splicing profile by RNA-seq. Heatmap of LSVs between the untreated and 20CTG **(A)** and 3′CTG **(B)** treated myotubes with significant ΔΨ. **(C)** Number of LSVs belonging to each of the categories and the respective schematic representation of the splicing modules. **(D)** Tree map depicting the three Gene Ontology categories found enriched in the list of LSVs found in the 20CTG treated myotubes. The size of the rectangles is proportional to the enrichment of the GO term. The numerals correspond to the number of genes from the input list involved in the GO term. The color gradient corresponds to the false discovery rate (FDR) where darker orange represents smaller FDR values. FDR cutoff was <0.05.

**FIGURE 6:**
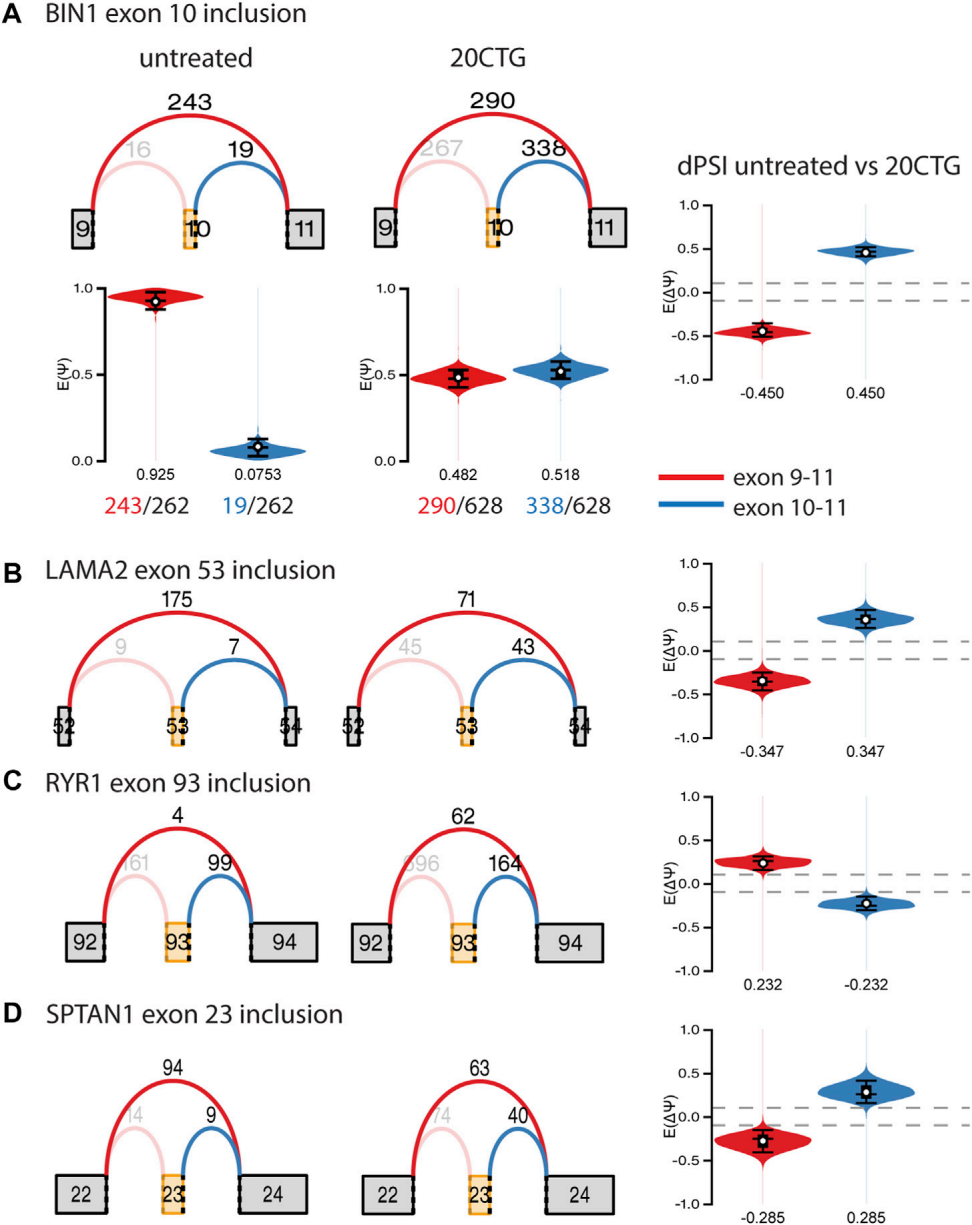
Graphs of selected local splice variants with junction read counts and violin plots showing the variation of percent of spliced in (ΔΨ/dPSI) values after treatment with 20CTG. **(A)** *BIN1* exon 10 inclusion. The red and blue arcs represent the junctions, and the numbers above the arcs are the junction read counts. The red and blue violins correspond to the PSI (E(Ψ)) or dPSI values of the respective junctions, where negative values correspond to increased differential inclusion in untreated condition compared with treated condition whereas a positive E [ΔΨ] denotes preference for treated vs. untreated. We show a detailed representation of BIN1 LSV as an example of how the dPSI is generated based on the individual PSI values obtained in each condition. **(B)** *LAMA2* exon 53 inclusion. **(C)** *RYR1* exon 93 inclusion. **(D)** *SPTAN1* exon 23 inclusion.

Subsequently, we analyzed the alternative splicing profile in AAV-treated versus untreated DM1 myotubes. We identified 96 and 247 local splicing variants (LSVs) in the 3′CTG and 20CTG treated myotubes, respectively. LSVs were subdivided into six modules of alternative splicing, namely alternative 3′, alternative intron, multi-exon spanning, alternate last exon, alternate first exon, and cassette (Figure 5C). LSVs with significant variation between untreated and treated conditions were plotted in heat maps and hierarchically clustered (Figures 5A, B). For each treatment, we can appreciate two discrete sets of LSVs with differential expression between treated and untreated DM1 myotubes. From the list of LSVs, we highlight a subset of genes that are known to have abnormal alternative splicing in DM1 and that showed a significant delta Percent Spliced Index (ΔΨ) after treatment with 20CTG (Figure 6) and 3′CTG (Supplementary Figure S7). Mis-splicing of *BIN1* exon 10 has been previously associated with T-tubule alterations which can partially explain the muscle weakness in DM1 (Fugier et al., 2011). We observed increased exon 10 inclusion after treatment with both constructs. We also identified the restoration of the splicing of *LAMA2, LDB3, MBNL1, FXR1,* and *SPTAN1* genes (Figure 6; Supplementary Figure S7).

To gain insight into the function of the genes in which alternative splicing was reversed by the AAV8. U7snRNA treatment, we performed Gene Ontology analysis on the lists of LSVs. We identified a list of enriched and representative GO terms that correlate with muscle development, structure, and function (Figure 5D) in the 20CTG treatment. GO results for 3′CTG treatment are shown in Supplementary Figure S8. This suggests that the splicing correction detected in the analysis above is relevant to the restoration of a normal muscle phenotype, and then functionally pertinent in the context of correction of DM1 pathology.

### Validation of spliceopathy correction by RT-PCR

**FIGURE 7:**
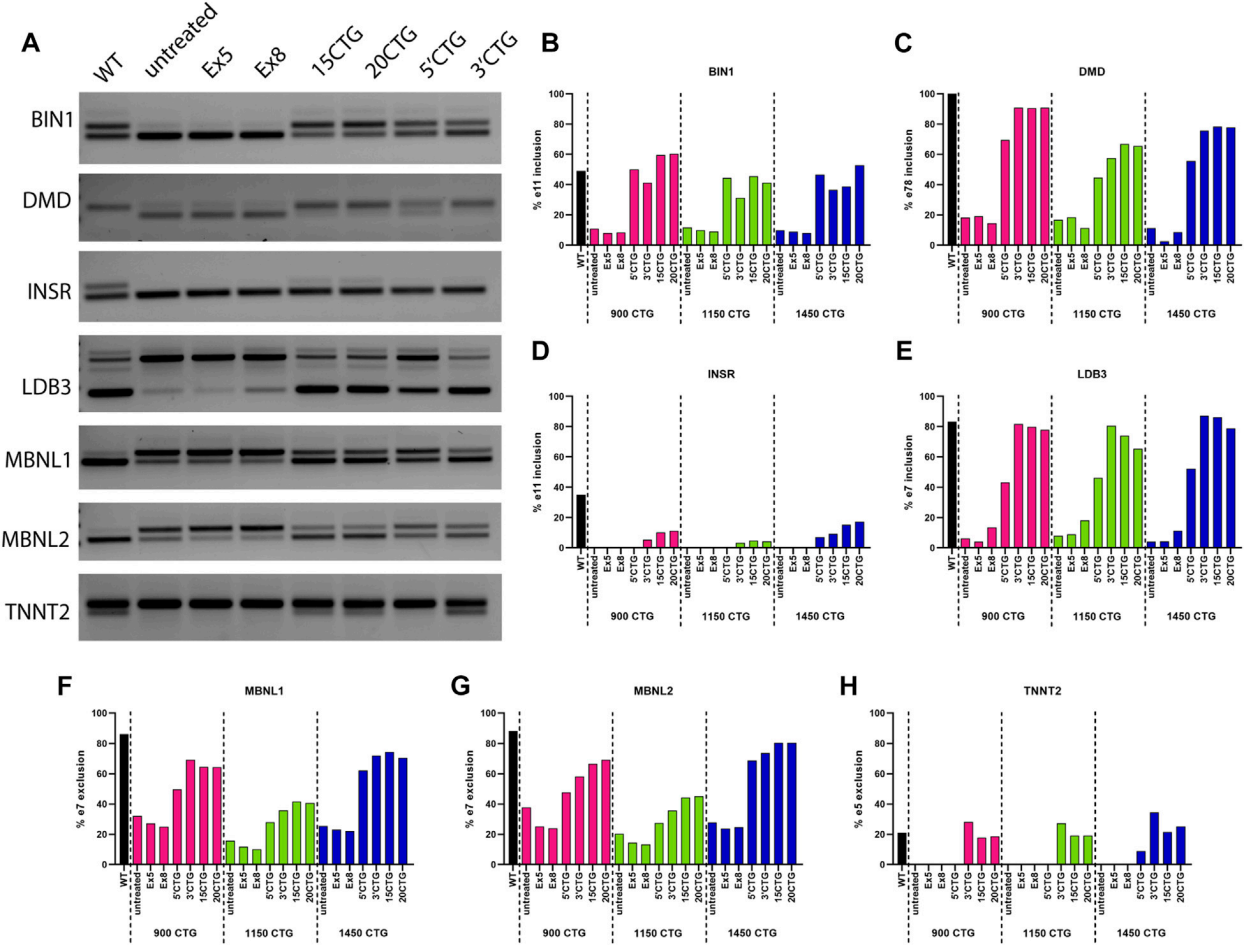
Validation of splicing pattern by RT-PCR. **(A)** Representative gel image of RT-PCR amplification of exons with differential splicing detected by RNA-seq and commonly altered in DM1. The first lane shows the expected normal pattern in healthy control cells. The subsequent lanes show the bands in 900CTG cell line untreated and treated with the different constructs tested in this study. Graphs **(B–H)** show the quantification of the band intensity of the included/excluded exons for each gene analyzed in the three cell lines. The constructs targeting the 3′UTR/CUG repeats region showed the best splicing correction, equivalent to normal levels.

To confirm the RNA sequencing findings on the correction of abnormal alternative splicing by the AAV8. U7snRNAs, we performed RT-PCR analysis on selected candidates. Moreover, to expand our findings on the effects of our constructs, we also analyzed the splicing pattern of additional classic DM1 biomarkers, specifically *DMD*, *INSR*, *MBNL1*, and *MBNL2* genes that were not detected by RNA-seq. This missed detection can be attributed to a lower number of reads, but PCR has a higher sensitivity for individual events and amplifies the signal as seen in the representative gel image (Figure 7A), showing the detection of alternatively spliced exons in seven genes implicated in DM1 pathology. A healthy control sample was included as a reference of the expected pattern of exon inclusion/exclusion, as well as an untreated DM1 sample and the respective samples treated with the different constructs tested in this study (Figure 7A). In panels B to H are plotted the quantification of the band intensity obtained for each DM1 cell line (Figure 7). The constructs targeting the 3′UTR/CUG repeat expansion region were the most successful in restoring the splicing pattern of all genes, with some of them reaching levels equivalent to the wild-type pattern.

## Discussion

The manifestation of multisystemic symptoms in DM1 can be explained by the pleiotropic effect caused by CTG expansion on a wide variety of genes which are developmentally regulated by alternative splicing. *DMPK* alleles containing an excessive number of CUG repeats form secondary structures that sequester MBNL proteins, and ultimately CELF1 phosphorylation is upregulated, favoring a fetal splicing profile of genes regulated by MBNL1. Thus, strategies aiming to restore normal levels of MBNL1 hold a promise to treat DM1. Considering this scenario, we designed multiple modified mU7snRNA to knock down DMPK expression or promote steric hindrance of the CUG repeats and prevent MBNL1 binding. The latter strategy proved to be very efficacious in reversing the main DM1 hallmarks, namely RNA *foci* and abnormal alternative splicing profile. In addition, for the first time, we used AAV vectors to deliver the U7snRNAs. AAV vectors have an excellent safety profile and there are a few products already available in the market to treat different genetic disorders.

The development of cellular models of tissues affected by DM1 that develop the main molecular hallmarks of the disease is critical to investigate the pathological mechanisms, as well as to test therapeutic compounds and/or vectors for gene therapy. Several *in vitro* models have been explored for DM1 as there is no perfect mouse model. Among them, primary myoblasts which can be derived from muscle biopsies, can reproduce *in vitro* some of the disease features and have helped study molecular mechanisms of DM1 (Furling et al., 2001; Dansithong et al., 2005; Holt et al., 2007; Loro et al., 2010; Botta et al., 2013). They have also been used to assess the efficacy of therapies (François et al., 2011; Jenquin et al., 2018; Reddy et al., 2019). Nonetheless, primary myoblasts cultures are a limited source of cells due to their restricted proliferation capacity. To circumvent this disadvantage, immortalized myoblast lines have been created and they were shown to reproduce the molecular features of DM1 (Pantic et al., 2016; Ludovic et al., 2017). However, they still require invasive muscle biopsies. Conversely, skin biopsies are easier to perform and are less harmful to patients. Fibroblasts derived from skin biopsies were used to establish DM1 induced pluripotent stem cells, which can differentiate into many different cell types and could potentially be used for cell therapy (Gao et al., 2016). Still, the derivation and maintenance of these cells are laborious, with variable efficiency, and the reprogramming process erases epigenetic markers that might be pertinent to the disease’s molecular mechanisms. As an alternative, immortalization and direct reprogramming of skin fibroblasts into myogenic lineages are relatively easy with the advantage of preserving the epigenome (Davis et al., 1987; O’Leary et al., 2010; Larsen et al., 2011; Provenzano et al., 2017).

Here, we obtained skin biopsies from patients with DM1, carrying different sizes of the CTG expansion, and successfully reprogrammed them into myoblasts, which have good myogenic potential *in vitro* and recapitulate molecular features observed in the DM1 muscles, like RNA *foci* accumulation and aberrant alternative splicing. *DMPK* expression increases with myoblast fusion *in vitro* (Furling et al., 2003) and RNA *foci* accumulate more in non-proliferating cells (Xia and Ashizawa, 2015). In addition, myotubes express muscle-specific proteins that are not expressed by myoblasts, with a transcriptional profile that resembles muscle samples. Therefore, differentiated myotubes are preferable to evaluate RNA *foci* accumulation instead of fibroblasts or myoblasts. While immortalized DM1 myoblasts behave in a similar way to primary myoblasts regarding their differentiation potential (Ludovic et al., 2017), it was shown that the restoration of *MYOD* levels in transdifferentiated DM1 fibroblasts was able to overcome the impaired differentiation (Larsen et al., 2011).

Next, we designed different antisense sequences targeting the splicing sites of exons 5 and 8 and the 3′UTR/CUG tract region to promote *DMPK* knockdown or steric blocking of the CUG repeats, respectively. We selected exons 5 and 8 because once skipped, an out-of-frame transcript is produced and likely degraded via the RNA surveillance pathway, and these two exons are present in all *DMPK* isoforms. This strategy is not allele-specific, meaning that both normal and expanded alleles are knocked down, and potentially DMPK protein levels would also be decreased. Though DMPK protein levels can be reduced by 50% in congenital DM1 (Furling et al., 2003), it is unclear whether DMPK haploinsufficiency is relevant to disease development. *Dmpk* knockout mouse models have been developed to address this question. The two first mouse strains obtained had mild cardiac conduction defects and mild myopathy (Jansen et al., 1996; Reddy et al., 1996). Later, a different *Dmpk* knockout mouse opposed these findings and had no phenotype (Carrell et al., 2016). Altogether, these studies indicate that DMPK may not be essential for muscle function in adults and knockdown strategies could be tolerable.

U7snRNAs have shown excellent efficiency in promoting exon skipping (Wein et al., 2022), so we designed modified mU7snRNAs to skip exons 5 or 8. After treating DM1 FM cells with AAV8, we verified the RNA *foci* quantity by FISH as an indication of decreased *DMPK* mRNA levels. Unfortunately, the reduction in the number of *foci* per nucleus was not significant, and neither the splicing pattern of the genes checked by RT-PCR was altered. Our results corroborate the findings of a recent publication in which antisense oligonucleotides promoting exon skipping were unsuccessful in rescuing DM1 hallmarks in fibroblasts (Stepniak-Konieczna et al., 2020).

Interference in the MBNL proteins interaction with the CUG repeats is a promising strategy to reverse the DM1 pathology (Wheeler et al., 2009). For that, modified antisense oligonucleotides have been tested both *in vitro* and *in vitro*. In the HSA^LR^, a mouse model that expresses a human actinin transgene containing CTG repeats in their skeletal muscle (Mankodi et al., 2000), different types of ASOs were able to promote RNA *foci* reduction, MBNL1 redistribution, correction of abnormal splicing, and myotonia improvement (Mulders et al., 2009; Wheeler et al., 2009; Leger et al., 2013; Wojtkowiak-Szlachcic et al., 2015; Hsieh et al., 2018; Klein et al., 2019). These results hold the promise to be effective to treat DM1 patients and a few drugs will be soon tested in clinical trials, but ASOs present some limitations that could potentially preclude their use in clinics, such as the requirement of high doses and long-life administration. This can be exemplified by a clinical trial in which an MOE gapmer (IONIS-DMPK-2.5Rx) aiming to reduce the expanded allele via RNase-H degradation pathway failed to show therapeutic benefits because of insufficient drug levels reaching the muscles (https://strongly.mda.org/ionis-reports-setback-dmpkrx-program-myotonic-dystrophy/). Therefore, new approaches to deliver ASOs are needed to achieve meaningful clinical outcomes.

Modified U7snRNAs can be used to deliver antisense sequences with several advantages over “naked” ASOs. The expression of U7snRNAs grants continuous expression of the therapeutic antisense sequence, ensuring long-term effect and minimizing the necessity of re-administration. U7snRNAs are protected from nuclease degradation due to stabilization by Sm and Lsm proteins. And delivery via AAV vectors favors an ample distribution of U7snRNAs to the targeted tissues, depending on the serotype selected (reviewed in [Lesman et al., 2021)].

Silencing of the expanded *DMPK* alleles by the use of a lentivirus expressing U7snRNA targeting the CUG repeat has been previously shown to knock down *DMPK* mRNA. It reduced the fraction of nuclei containing *foci* and restored the splicing of *BIN1*, *DMD*, and *LDB3* genes in DM1 muscle cells (François et al., 2011). Despite being promising this approach does not seem to have been explored in animal models potentially due to safety issues with the use of lentivirus. To further explore this strategy, we designed three new antisense sequences to target the 3′UTR/CUG tract region and included a construct similar to the one used in the previous study with 15 CTG repeats. These constructs were delivered thanks to the use of AAVs, which are vectors currently in human clinical trials. Our 20CTG construct has the advantage to be even more specific and it covers more CUG repeats at once. The 5′CTG and 3′CTG constructs have further specificity to *DMPK* transcripts because they also target the 3′UTR sequences next to the CUG tract, minimizing potential off-targets. Furthermore, instead of using lentivirus (LVs) as a delivery vector, we utilized adeno-associated viruses that present a series of advantages in terms of safety over LVs. AAVs have an extremely low rate of genome integration, reducing the risks of mutation insertion or disruption of coding genes. Instead, AAV genomes persist as episomal chromatin in the nucleus of transduced cells. Of note, the transgene expression persists in the long term. And most importantly, different studies have shown that AAVs are non-toxic in nonhuman primates (Gushchina et al., 2021) nor in humans, as exampled by the FDA-approved drug onasemnogene abeparvovec to treat spinal muscular atrophy (Mendell et al., 2021).

After the treatment with the AAV8. U7snRNAs targeting the CUG repeats, there was a significant decrease in the number of *foci* per nucleus, accompanied by MBNL1 re-localization. Improved myogenic differentiation was also observed. To further explore the effects of these two observations, we investigated the impact of the U7snRNAs 20CTG and 3′CTG constructs on the transcriptome by RNA sequencing, which had not been explored before. Former publications on the DM1 transcriptome have revealed specific signatures highly correlated with muscle strength (Wang E. T. et al., 2019). Transcriptome analysis of primary DM1 myoblasts and myotubes showed mis-regulation of proliferation, extracellular matrix, and cytoskeleton, which will ultimately compromise myoblast fusion and differentiation, contributing to the DM1 phenotype (Todorow et al., 2021). In our study, differential expression analysis revealed the de-regulation of approximately a hundred genes, which are mainly related to immune response as indicated by Gene Ontology enrichment analysis. As expected, the enrichment of immune response categories is probably a response to the AAV8 particles and not DM1-specific. Immune responses to the AAV capsid, both innate and adaptative, are well documented in the literature (Martino and Markusic, 2020; Ronzitti et al., 2020; Ertl, 2021). Considering the cells were harvested only 9 days after AAV exposure, the expression of such genes is still activated and maybe is masking transcriptome changes that could occur in response to the MBNL1 release. Differently from the previous publication (François et al., 2011), overall *DMPK* expression was unaffected by the treatment, suggesting that RNA *foci* decrease is not occurring via mRNA decay. We cannot establish either whether our constructs can target the normal alleles since the cells utilized do not have SNPs that could distinguish both alleles. Thus, future studies may be needed to clarify these questions and determine the exact mechanism.

Splicing alterations are known to be more distinctive and broader in the DM1 transcriptome (Nakamori et al., 2013; Wang D. et al., 2019). Thus, we focused on the alternative splicing profile upon the treatment with the 20CTG and 3′CTG constructs. We found an evident shift in the splicing pattern of many exons before and after treatment (Figures 5A, B). Some of the cassette exons identified in our sample data were also recognized as splicing candidates in DM1 muscle biopsies (Nakamori et al., 2013), reinforcing the significance of these splicing events in DM1. Moreover, this also suggests that the FM cell model has an expression profile that partially overlaps with the mature muscle profile, confirming the reliability of this model as a tool to test therapies *in vitro*. Gene Ontology analysis confirms the pertinency of these genes by the enrichment of several terms correlated with muscle development, structure, and function, such as “muscle organ development,” “cytoskeleton organization,” and “sarcomere”. Finally, by RT-PCR we were able to confirm the splicing profile of selected candidates and extend the analysis to genes not captured by RNA-seq. Altogether, our results show that the U7snRNAs are effectively delivering antisense sequences, which are binding the CUG repeats, displacing MBNL1 protein, and finally leading to spliceopathy correction of genes implicated in DM1 muscle phenotype.

In conclusion, we have developed DM1 FM cell lines that have the advantage to be derived from skin biopsies, which are less invasive compared to muscle biopsy, extended proliferation *in vitro*, and simple conversion into mature myotubes in a short time frame. FM lines recapitulate two of the main cellular and molecular DM1 features in muscle cells, RNA *foci,* and altered alternative splicing. We employed two different strategies aiming to reverse the DM1 pathology: one based on exon skipping to produce an out-of-frame transcript and consequent mRNA degradation, and a steric hindrance one, through competitive binding with the CUG repeats. The latter strategy proved to be more efficacious, as evidenced by RNA *foci* decrease and MBNL1 re-localization. Our findings further extend previous studies by exploring the global transcriptome, showing a wide correction of spliceopathy in different DM1 cell lines, and by delivering U7snRNAs by AAV vectors, which have a safer profile for *in vivo* studies. While cell models are excellent tools to screen therapeutics, they still have limitations. Therefore, studies on DM1 animal models will soon be conducted to investigate the efficacy of our best candidates *in vivo*.

## Data Availability

The data presented in the study are deposited in the Gene Expression Omnibus (GEO-NCBI) repository, accession number GSE233947.

## Supplementary material

SUPPLEMENTARY FIGURE S1 Myosin heavy chain expression in FM cell lines. **(A)** DM1 cell lines obtained with different CTG expansion lengths. Differentiation of FM lines was confirmed by myosin heavy chain expression (green). Cell lines with fewer repeats tend to show better myogenic differentiation by forming larger myotubes. Nuclei in blue. Scale bar = 100 µm. **(B)** Undifferentiated fibroblasts and myoblasts do not express myosin heavy chain protein. Scale bar = 20 µm.

SUPPLEMENTARY FIGURE S2 RNA *foci* and MBNL expression in healthy control cells. **(A)** RNA FISH on cells containing a normal range of CTG repeats in DMPK. **(B)** Quantification of RNA *foci* showing virtual absence of *foci*. Around 6,000 nuclei were measured. **(C)** MBNL1 staining (green) combined with RNA FISH (red) showing diffuse localization of MBNL1 in the nucleus of healthy cells.

SUPPLEMENTARY FIGURE S3 Exon skipping efficiency and myoblast fusion. RT-PCR of exons 5 **(A)** and 8 **(B)** to detect exon skipping efficiency. No skipping of exon 5 was observed. Only construct Ex8#1 showed around 30% of skipping. **(C)** Counting of distinct nuclear domains in the three DM1 cell lines showed a reduction in the number of domains, indicating more large nuclei clusters, which is an approximate measurement of myoblast fusion.

SUPPLEMENTARY FIGURE S4 Zoomed in images of individual nuclei to highlight the reduction in the number of RNA *foci* after treatment with AAV.U7snRNAs.

SUPPLEMENTARY FIGURE S5 MBNL1 is released from RNA *foci* and re-localizes in the nucleoplasm. RNA FISH (red) combined with immunofluorescence staining for MBNL1 (green) on **1150CTG** DM1 cell line, before and after treatment with AAV8.U7snRNAs targeting the 3’UTR/CUG tract region. In untreated cells, MBNL1 staining shows a dotted pattern and co-localizes with RNA *foci*, as seen in the merged image in yellowish color. After treatment, the number of RNA *foci* reduces dramatically, releasing MBNL1 which now shows a diffuse localization in the nucleus. Scale bar = 20 µm.

SUPPLEMENTARY FIGURE S6 MBNL1 is released from RNA *foci* and re-localizes in the nucleoplasm. RNA FISH (red) combined with immunofluorescence staining for MBNL1 (green) on **1450CTG** DM1 cell line, before and after treatment with AAV8.U7snRNAs targeting the 3’UTR/CUG tract region. In untreated cells, MBNL1 staining shows a dotted pattern and co-localizes with RNA *foci*, as seen in the merged image. After treatment, the number of RNA *foci* reduces dramatically, releasing MBNL1 which now shows a diffuse localization in the nucleus. Scale bar = 20 µm.

SUPPLEMENTARY FIGURE S7 Graphs of selected local splice variants with junction read counts and violin plots showing the variation of percent of spliced in (ΔΨ/dPSI) values after treatment with 3’CTG. **(A)** *BIN1* exon 10 inclusion. The red and blue arcs represent the junctions, and the numbers above the arcs are the junction read counts. The red and blue violins correspond to dPSI values of the respective junctions, where negative values correspond to increased differential inclusion in untreated condition compared with treated condition whereas a positive E[ΔΨ] denotes preference for treated vs untreated. **(B)** *FXR1* exon 16 inclusion. **(C)** *MBNL1* exon 7 exclusion. **(D)** *SPTAN1* exon 23 inclusion. **(E)** *LDB3* exon 8 inclusion.

SUPPLEMENTARY FIGURE S8 Tree map depicting the three Gene Ontology categories found enriched in the list of LSVs found in the 3’CTG treated myotubes. The size of the rectangles is proportional to the enrichment of the GO term. The numerals correspond to the number of genes from the input list involved in the GO term. The color gradient corresponds to the false discovery rate (FDR) where darker orange represents smaller FDR values. FDR cutoff was <0.05.

## References

[B1] Ait Benichou S. Jauvin D. De Serres-Bérard T. Pierre M. Ling K. K. Bennett C. F. (2022). Antisense oligonucleotides as a potential treatment for brain deficits observed in myotonic dystrophy type 1. Gene Ther. 29, 698–709. 10.1038/S41434-022-00316-7 35075265 PMC9750879

[B2] Botta A. Malena A. Loro E. Del Moro G. Suman M. Pantic B. (2013). Altered Ca2+ homeostasis and endoplasmic reticulum stress in Myotonic Dystrophy type 1 muscle cells. Genes (Basel) 4, 275–292. 10.3390/genes4020275 24705164 PMC3899969

[B3] Carrell S. T. Carrell E. M. Auerbach D. Pandey S. K. Bennett C. F. Dirksen R. T. (2016). Dmpk gene deletion or antisense knockdown does not compromise cardiac or skeletal muscle function in mice. Hum. Mol. Genet. 25, 4328–4338. 10.1093/hmg/ddw266 27522499 PMC5291200

[B4] Dansithong W. Paul S. Comai L. Reddy S. (2005). MBNL1 is the primary determinant of focus formation and aberrant insulin receptor splicing in DM1. J. Biol. Chem. 280, 5773–5780. 10.1074/jbc.M410781200 15546872

[B5] Davis B. M. Mccurrach M. E. Taneja K. L. Singer R. H. Housman D. E. (1997). Expansion of a CUG trinucleotide repeat in the 3′ untranslated region of myotonic dystrophy protein kinase transcripts results in nuclear retention of transcripts. Proc. Natl. Acad. Sci. U. S. A. 94, 7388–7393. 10.1073/pnas.94.14.7388 9207101 PMC23831

[B6] Davis R. L. Weintraub H. Lassar A. B. (1987). Expression of a single transfected cDNA converts fibroblasts to myoblasts. Cell 51, 987–1000. 10.1016/0092-8674(87)90585-X 3690668

[B7] Dobin A. Davis C. A. Schlesinger F. Drenkow J. Zaleski C. Jha S. (2013). Star: Ultrafast universal RNA-seq aligner. Bioinformatics 29, 15–21. 10.1093/bioinformatics/bts635 23104886 PMC3530905

[B8] Ertl H. C. J. (2021). T cell-mediated immune responses to AAV and AAV vectors. Front. Immunol. 12, 666666. 10.3389/FIMMU.2021.666666 33927727 PMC8076552

[B9] François V. Klein A. F. Beley C. Jollet A. Lemercier C. Garcia L. (2011). Selective silencing of mutated mRNAs in DM1 by using modified hU7-snRNAs. Nat. Struct. Mol. Biol. 18, 85–87. 10.1038/nsmb.1958 21186365

[B10] Fu Y. H. Pizzuti A. Fenwick R. G. King J. Rajnarayan S. Dunne P. W. (1992). An unstable triplet repeat in a gene related to myotonic muscular dystrophy. Science 255, 1256–1258. 10.1126/science.1546326 1546326

[B11] Fugier C. Klein A. F. Hammer C. Vassilopoulos S. Ivarsson Y. Toussaint A. (2011). Misregulated alternative splicing of BIN1 is associated with T tubule alterations and muscle weakness in myotonic dystrophy. Nat. Med. 17, 720–725. 10.1038/nm.2374 21623381

[B12] Furling D. Coiffier L. Mouly V. Barbet J. P. Lacau J. Taneja K. (2001). Defective satellite cells in congenital myotonic dystrophy. Hum. Mol. Genet. 10, 2079–2087. 10.1093/hmg/10.19.2079 11590125

[B13] Furling D. Lam L. T. Agbulut O. Butler-Browne G. S. Morris G. E. (2003). Changes in myotonic dystrophy protein kinase levels and muscle development in congenital myotonic dystrophy. Am. J. Pathol. 162, 1001. 10.1016/S0002-9440(10)63894-1 12598332 PMC1868110

[B14] Gao Y. Guo X. Santostefano K. Wang Y. Reid T. Zeng D. (2016). Genome therapy of myotonic dystrophy type 1 iPS cells for development of autologous stem cell therapy. Mol. Ther. 24, 1378–1387. 10.1038/mt.2016.97 27203440 PMC5023370

[B15] González-Barriga A. Mulders S. A. M. Van De Giessen J. Hooijer J. D. Bijl S. Van Kessel I. D. G. (2013). Design and analysis of effects of triplet repeat oligonucleotides in cell models for myotonic dystrophy. Mol. Ther. - Nucleic Acids 2, e81. 10.1038/mtna.2013.9 23511335 PMC3615819

[B16] Gushchina L. V. Frair E. C. Rohan N. Bradley A. J. Simmons T. R. Chavan H. D. (2021). Lack of toxicity in nonhuman primates receiving clinically relevant doses of an AAV9.U7snRNA vector designed to induce DMD exon 2 skipping. Hum. Gene Ther. 32, 882–894. 10.1089/HUM.2020.286 33406986 PMC10112461

[B17] Holt I. Mittal S. Furling D. Butler-Browne G. S. Brook J. D. Morris G. E. (2007). Defective mRNA in myotonic dystrophy accumulates at the periphery of nuclear splicing speckles. Genes Cells 12, 1035–1048. 10.1111/j.1365-2443.2007.01112.x 17825047

[B18] Hsieh W. C. Bahal R. Thadke S. A. Bhatt K. Sobczak K. Thornton C. (2018). Design of a “mini” nucleic acid probe for cooperative binding of an RNA-repeated transcript associated with myotonic dystrophy type 1. Biochemistry 57, 907–911. 10.1021/ACS.BIOCHEM.7B01239 29334465 PMC6091549

[B19] Hu N. Kim E. Antoury L. Li J. González-Pérez P. Rutkove S. B. (2021). Antisense oligonucleotide and adjuvant exercise therapy reverse fatigue in old mice with myotonic dystrophy. Mol. Ther. - Nucleic Acids 23, 393–405. 10.1016/j.omtn.2020.11.014 33473325 PMC7787993

[B20] Izzo M. Battistini J. Provenzano C. Martelli F. Cardinali B. Falcone G. (2022). Molecular therapies for myotonic dystrophy type 1: From small drugs to gene editing. Int. J. Mol. Sci. 23, 4622. 10.3390/IJMS23094622 35563013 PMC9101876

[B21] Jansen G. Groenen P. J. T. A. Bächner D. Jap P. H. K. Coerwinkel M. Oerlemans F. (1996). Abnormal myotonic dystrophy protein kinase levels produce only mild myopathy in mice. Nat. Genet. 13, 316–324. 10.1038/NG0796-316 8673131

[B22] Jauvin D. Chrétien J. Pandey S. K. Martineau L. Revillod L. Bassez G. (2017). Targeting DMPK with antisense oligonucleotide improves muscle strength in myotonic dystrophy type 1 mice. Mol. Ther. - Nucleic Acids 7, 465–474. 10.1016/j.omtn.2017.05.007 28624222 PMC5453865

[B23] Jenquin J. R. Coonrod L. A. Silverglate Q. A. Pellitier N. A. Hale M. A. Xia G. (2018). Furamidine rescues myotonic dystrophy type i associated mis-splicing through multiple mechanisms. ACS Chem. Biol. 13, 2708–2718. 10.1021/acschembio.8b00646 30118588 PMC6343479

[B24] Johnson N. E. Butterfield R. J. Mayne K. Newcomb T. Imburgia C. Dunn D. (2021). Population based prevalence of myotonic dystrophy type 1 using genetic analysis of state-wide blood screening program. Neurology 96, e1045–e1053. 10.1212/WNL.0000000000011425 33472919 PMC8055332

[B25] Klein A. F. Varela M. A. Arandel L. Holland A. Naouar N. Arzumanov A. (2019). Peptide-conjugated oligonucleotides evoke long-lasting myotonic dystrophy correction in patient-derived cells and mice. J. Clin. Invest. 129, 4739–4744. 10.1172/JCI128205 31479430 PMC6819114

[B26] Kuyumcu-Martinez N. M. Wang G. S. Cooper T. A. (2007). Increased steady-state levels of CUGBP1 in myotonic dystrophy 1 are due to PKC-mediated hyperphosphorylation. Mol. Cell 28, 68–78. 10.1016/j.molcel.2007.07.027 17936705 PMC2083558

[B27] Larsen J. Pettersson O. J. Jakobsen M. Thomsen R. Pedersen C. B. Hertz J. M. (2011). Myoblasts generated by lentiviral mediated MyoD transduction of myotonic dystrophy type 1 (DM1) fibroblasts can be used for assays of therapeutic molecules. BMC Res. Notes 4, 490. 10.1186/1756-0500-4-490 22078098 PMC3226528

[B28] Leger A. J. Mosquea L. M. Clayton N. P. Wu I. H. Weeden T. Nelson C. A. (2013). Systemic delivery of a peptide-linked morpholino oligonucleotide neutralizes mutant RNA toxicity in a mouse model of myotonic dystrophy. Nucleic Acid. Ther. 23, 109–117. 10.1089/nat.2012.0404 23308382

[B29] Lesman D. Rodriguez Y. Rajakumar D. Wein N. (2021). U7 snRNA, a small RNA with a big impact in gene therapy. Hum. Gene Ther. 32, 1317–1329. 10.1089/HUM.2021.047 34139889

[B30] Liao Y. Smyth G. K. Shi W. (2014). featureCounts: an efficient general purpose program for assigning sequence reads to genomic features. Bioinformatics 30, 923–930. 10.1093/BIOINFORMATICS/BTT656 24227677

[B31] Liao Y. Wang J. Jaehnig E. J. Shi Z. Zhang B. (2019). WebGestalt 2019: Gene set analysis toolkit with revamped UIs and APIs. Nucleic Acids Res. 47, W199–W205. 10.1093/NAR/GKZ401 31114916 PMC6602449

[B32] Loro E. Rinaldi F. Malena A. Masiero E. Novelli G. Angelini C. (2010). Normal myogenesis and increased apoptosis in myotonic dystrophy type-1 muscle cells. Cell Death Differ. 17, 1315–1324. 10.1038/cdd.2010.33 20431600

[B33] Ludovic A. Micaela P.-E. Magdalena M. Audrey B. Damily D. D. D. Naïra N. (2017). Immortalized human myotonic dystrophy muscle cell lines to assess therapeutic compounds. Dis. Model. Mech. 10, 487–497. 10.1242/dmm.027367 28188264 PMC5399563

[B34] Magoč T. Salzberg S. L. (2011). Flash: Fast length adjustment of short reads to improve genome assemblies. Bioinformatics 27, 2957–2963. 10.1093/bioinformatics/btr507 21903629 PMC3198573

[B35] Mahadevan M. Tsilfidis C. Sabourin L. Shutler G. Amemiya C. Jansen G. (1992). Myotonic dystrophy mutation: An unstable CTG repeat in the 3′ untranslated region of the gene. Science 255, 1253–1255. 10.1126/science.1546325 1546325

[B36] Mankodi A. Logigian E. Callahan L. McClain C. White R. Henderson D. (2000). Myotonic dystrophy in transgenic mice expressing an expanded CUG repeat. Science 289, 1769–1772. 10.1126/science.289.5485.1769 10976074

[B37] Mankodi A. Takahashi M. P. Jiang H. Beck C. L. Bowers W. J. Moxley R. T. (2002). Expanded CUG repeats trigger aberrant splicing of ClC-1 chloride channel pre-mRNA and hyperexcitability of skeletal muscle in myotonic dystrophy. Mol. Cell 10, 35–44. 10.1016/S1097-2765(02)00563-4 12150905

[B38] Martino A. T. Markusic D. M. (2020). Immune response mechanisms against AAV vectors in animal models. Mol. Ther. Methods Clin. Dev. 17, 198. 10.1016/J.OMTM.2019.12.008 31970198 PMC6965504

[B39] Mendell J. R. Al-Zaidy S. A. Lehman K. J. McColly M. Lowes L. P. Alfano L. N. (2021). Five-year extension results of the phase 1 START trial of onasemnogene abeparvovec in spinal muscular atrophy. JAMA Neurol. 78, 834–841. 10.1001/JAMANEUROL.2021.1272 33999158 PMC8129901

[B40] Mulders S. A. M. Van Den Broek W. J. A. A. Wheeler T. M. Croes H. J. E. Van Kuik-Romeijn P. De Kimpe S. J. (2009). Triplet-repeat oligonucleotide-mediated reversal of RNA toxicity in myotonic dystrophy. Proc. Natl. Acad. Sci. U. S. A. 106, 13915–13920. 10.1073/pnas.0905780106 19667189 PMC2728995

[B41] Nakamori M. Gourdon G. Thornton C. A. (2011). Stabilization of expanded (CTG) (CAG) repeats by antisense oligonucleotides. Mol. Ther. 19, 2222–2227. 10.1038/mt.2011.191 21971425 PMC3242663

[B42] Nakamori M. Sobczak K. Puwanant A. Welle S. Eichinger K. Pandya S. (2013). Splicing biomarkers of disease severity in myotonic dystrophy. Ann. Neurol. 74, 862–872. 10.1002/ana.23992 23929620 PMC4099006

[B43] O’Leary D. A. Vargas L. Sharif O. Garcia M. E. Sigal Y. J. Chow S. K. (2010). Hts-compatible patient-derived cell-based assay to identify small molecule modulators of aberrant splicing in myotonic dystrophy type 1. Curr. Chem. Genomics 4, 9–18. 10.2174/1875397301004010009 20502647 PMC2874217

[B44] Pandey S. K. Wheeler T. M. Justice S. L. Kim A. Younis H. S. Gattis D. (2015). Identification and characterization of modified antisense oligonucleotides targeting DMPK in mice and nonhuman primates for the treatment of myotonic dystrophy type 1. J. Pharmacol. Exp. Ther. 355, 329–340. 10.1124/JPET.115.226969 26330536 PMC4613955

[B45] Pantic B. Borgia D. Giunco S. Malena A. Kiyono T. Salvatori S. (2016). Reliable and versatile immortal muscle cell models from healthy and myotonic dystrophy type 1 primary human myoblasts. Exp. Cell Res. 342, 39–51. 10.1016/j.yexcr.2016.02.013 26905645

[B46] Provenzano C. Cappella M. Valaperta R. Cardani R. Meola G. Martelli F. (2017). CRISPR/Cas9-Mediated deletion of CTG expansions recovers normal phenotype in myogenic cells derived from myotonic dystrophy 1 patients. Mol. Ther. - Nucleic Acids 9, 337–348. 10.1016/j.omtn.2017.10.006 29246312 PMC5684470

[B47] Rau F. Lainé J. Ramanoudjame L. Ferry A. Arandel L. Delalande O. (2015). Abnormal splicing switch of DMD’s penultimate exon compromises muscle fibre maintenance in myotonic dystrophy. Nat. Commun. 6, 7205. 10.1038/ncomms8205 26018658 PMC4458869

[B48] Reddy K. Jenquin J. R. McConnell O. L. Cleary J. D. Richardson J. I. Pinto B. S. (2019). A CTG repeat-selective chemical screen identifies microtubule inhibitors as selective modulators of toxic CUG RNA levels. Proc. Natl. Acad. Sci. 116, 20991–21000. 10.1073/pnas.1901893116 31570586 PMC6800345

[B49] Reddy S. Smith D. B. J. Rich M. M. Leferovich J. M. Reilly P. Davis B. M. (1996). Mice lacking the myotonic dystrophy protein kinase develop a late onset progressive myopathy. Nat. Genet. 13, 325–335. 10.1038/NG0796-325 8673132

[B50] Ronzitti G. Gross D. A. Mingozzi F. (2020). Human immune responses to adeno-associated virus (AAV) vectors. Front. Immunol. 11, 670. 10.3389/FIMMU.2020.00670/BIBTEX 32362898 PMC7181373

[B51] Sicot G. Gourdon G. Gomes-Pereira M. (2011). Myotonic dystrophy, when simple repeats reveal complex pathogenic entities: New findings and future challenges. Hum. Mol. Genet. 20, R116–R123. 10.1093/hmg/ddr343 21821673

[B52] Smyth G. K. Ritchie M. E. Law C. W. Alhamdoosh M. Su S. Dong X. (2018). RNA-seq analysis is easy as 1-2-3 with limma, Glimma and edgeR. F1000Research 5, ISCB Comm J-1408. 10.12688/f1000research.9005.3 PMC4937821 27441086

[B53] Stepniak-Konieczna E. Konieczny P. Cywoniuk P. Dluzewska J. Sobczak K. (2020). AON-induced splice-switching and DMPK pre-mRNA degradation as potential therapeutic approaches for Myotonic Dystrophy type 1. Nucleic Acids Res. 48, 2531–2543. 10.1093/nar/gkaa007 31965181 PMC7049696

[B54] Taneja K. L. McCurrach M. Schalling M. Housman D. Singer R. H. (1995). Foci of trinucleotide repeat transcripts in nuclei of myotonic dystrophy cells and tissues. J. Cell Biol. 128, 995–1002. 10.1083/jcb.128.6.995 7896884 PMC2120416

[B55] Thornton C. A. (2014). Myotonic dystrophy. Neurol. Clin. 32, 705–719. 10.1016/j.ncl.2014.04.011 25037086 PMC4105852

[B56] Todorow V. Hintze S. Kerr A. R. W. Hehr A. Schoser B. Meinke P. (2021). Transcriptome analysis in a primary human muscle cell differentiation model for myotonic dystrophy type 1. Int. J. Mol. Sci. 22, 8607. 10.3390/ijms22168607 34445314 PMC8395314

[B57] Vaquero-Garcia J. Barrera A. Gazzara M. R. González-Vallinas J. Lahens N. F. Hogenesch J. B. (2016). A new view of transcriptome complexity and regulation through the lens of local splicing variations. Elife 5, e11752. 10.7554/eLife.11752 26829591 PMC4801060

[B58] Wang D. Tai P. W. L. Gao G. (2019a). Adeno-associated virus vector as a platform for gene therapy delivery. Nat. Rev. Drug Discov. 18, 358. 10.1038/S41573-019-0012-9 30710128 PMC6927556

[B59] Wang E. T. Cody N. A. L. Jog S. Biancolella M. Wang T. T. Treacy D. J. (2012). Transcriptome-wide regulation of pre-mRNA splicing and mRNA localization by muscleblind proteins. Cell 150, 710–724. 10.1016/j.cell.2012.06.041 22901804 PMC3428802

[B60] Wang E. T. Treacy D. Eichinger K. Struck A. Estabrook J. Olafson H. (2019b). Transcriptome alterations in myotonic dystrophy skeletal muscle and heart. Hum. Mol. Genet. 28, 1312–1321. 10.1093/HMG/DDY432 30561649 PMC6452195

[B61] Wein N. Vetter T. A. Vulin A. Simmons T. R. Frair E. C. Bradley A. J. (2022). Systemic delivery of an AAV9 exon-skipping vector significantly improves or prevents features of Duchenne muscular dystrophy in the Dup2 mouse. Mol. Ther. - Methods Clin. Dev. 26, 279–293. 10.1016/j.omtm.2022.07.005 35949298 PMC9356240

[B62] Wheeler T. M. Leger A. J. Pandey S. K. Mac Leod A. R. Wheeler T. M. Cheng S. H. (2012). Targeting nuclear RNA for *in vivo* correction of myotonic dystrophy. Nature 488, 111–115. 10.1038/nature11362 22859208 PMC4221572

[B63] Wheeler T. M. Sobczak K. Lueck J. D. Osborne R. J. Lin X. Dirksen R. T. (2009). Reversal of RNA dominance by displacement of protein sequestered on triplet repeat RNA. Science 325, 336–339. 10.1126/science.1173110 19608921 PMC4109973

[B64] Wojtkowiak-Szlachcic A. Taylor K. Stepniak-Konieczna E. Sznajder L. J. Mykowska A. Sroka J. (2015). Short antisense-locked nucleic acids (all-LNAs) correct alternative splicing abnormalities in myotonic dystrophy. Nucleic Acids Res. 43, 3318–3331. 10.1093/nar/gkv163 25753670 PMC4381072

[B65] Xia G. Ashizawa T. (2015). Dynamic changes of nuclear RNA foci in proliferating DM1 cells. Histochem. Cell Biol. 143, 557–564. 10.1007/s00418-015-1315-5 25715678 PMC4439307

[B66] Yadava R. S. Yu Q. Mandal M. Rigo F. Bennett C. F. Mahadevan M. S. (2020). Systemic therapy in an RNA toxicity mouse model with an antisense oligonucleotide therapy targeting a non-CUG sequence within the DMPK 3 UTR RNA. Hum. Mol. Genet. 29, 1440–1453. 10.1093/hmg/ddaa060 32242217 PMC7268549

[B67] Yamamoto T. Miura A. Itoh K. Takeshima Y. Nishio H. (2019). RNA sequencing reveals abnormal LDB3 splicing in sudden cardiac death. Forensic Sci. Int. 302, 109906. 10.1016/j.forsciint.2019.109906 31419596

