# Supplementary figures and images for: Promising AAV.U7snRNAs vectors targeting DMPK improve DM1 hallmarks in patient-derived cell lines
Source: Front Cell Dev Biol. 2023 Jun 15;11:1181040. doi: 10.3389/fcell.2023.1181040 (PMC10309041; doi:10.3389/fcell.2023.1181040)

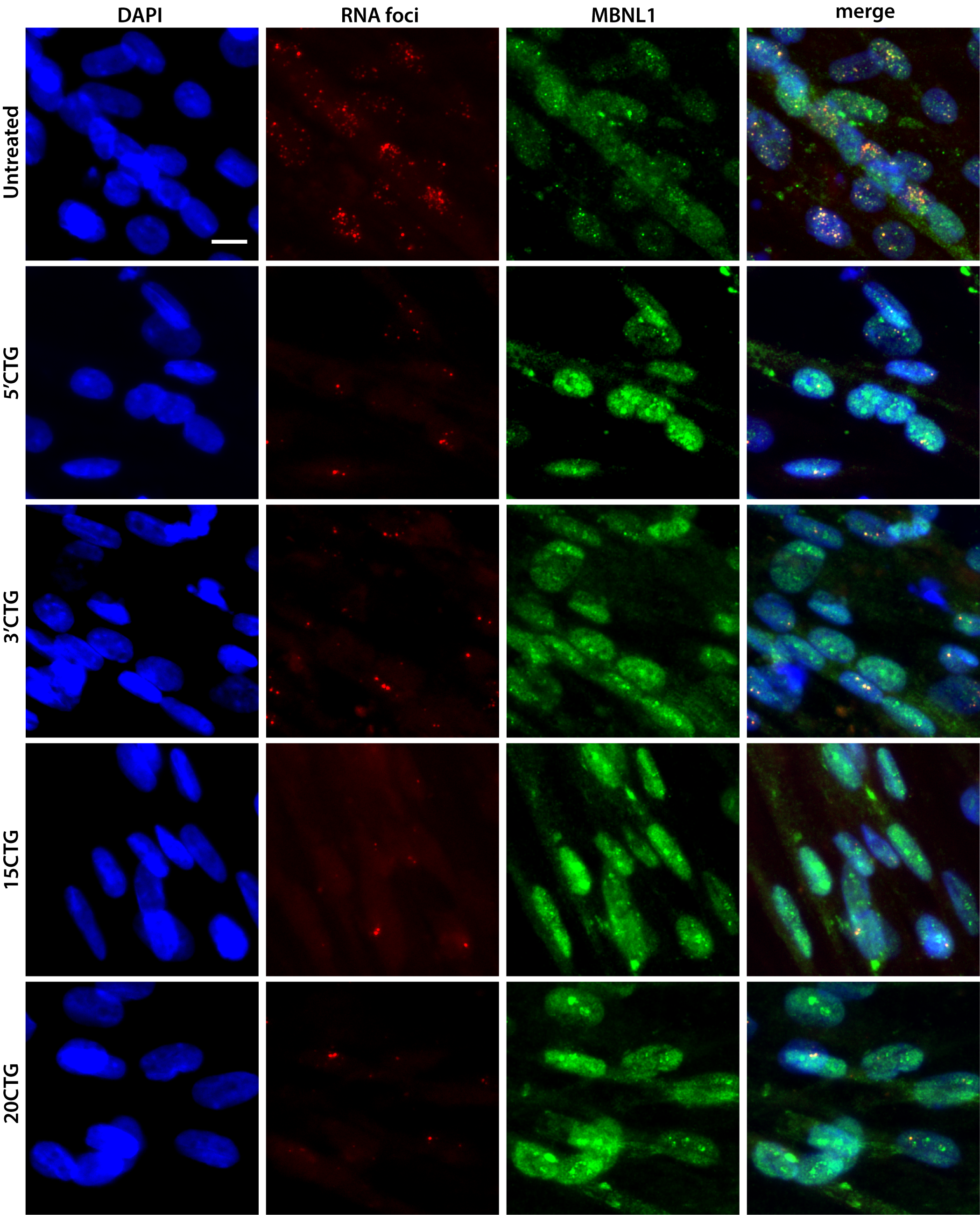

Supplement: Supplementary file 2 [file Image6.TIF]

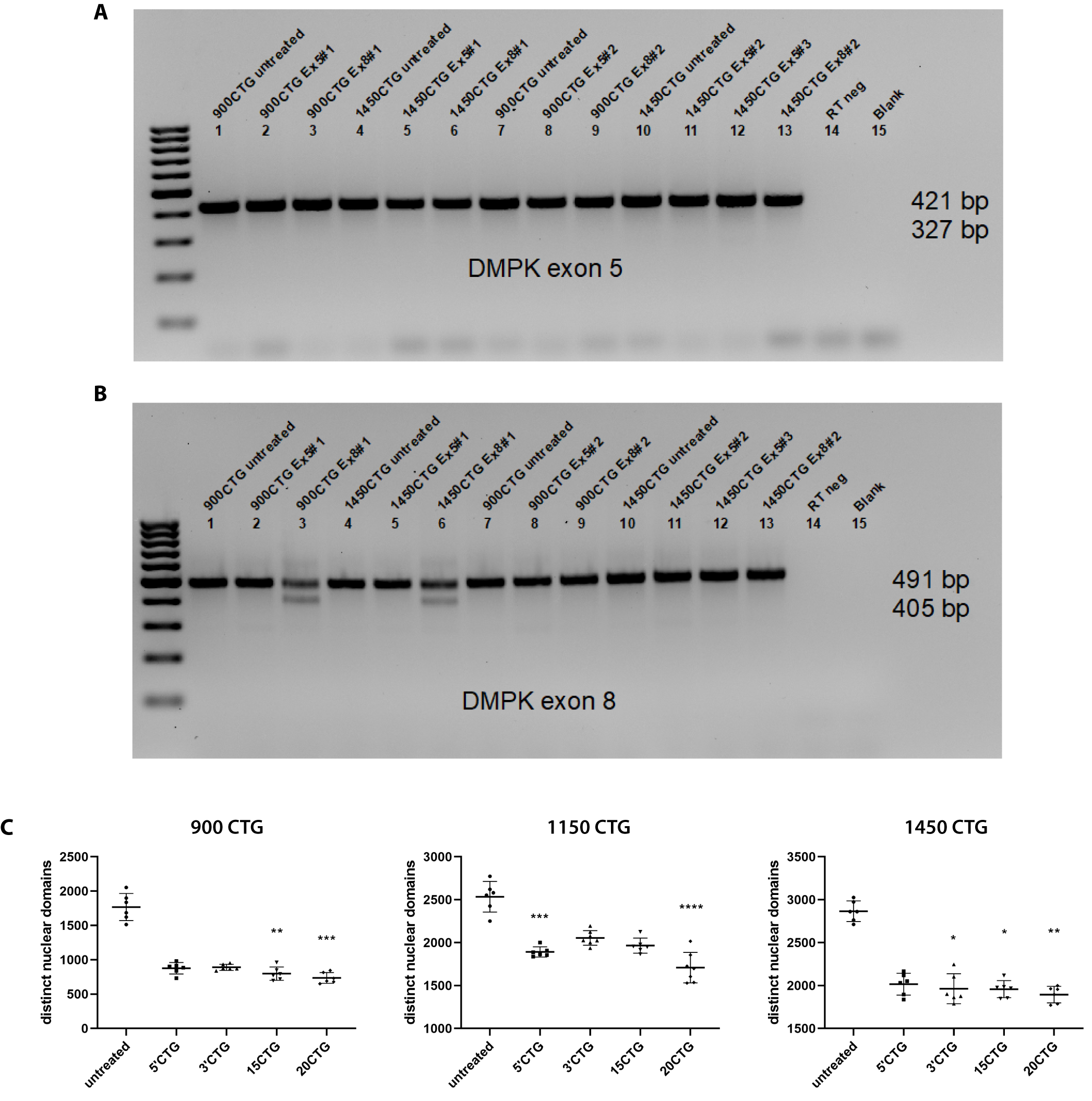

Supplement: Supplementary file 4 [file Image3.TIF]

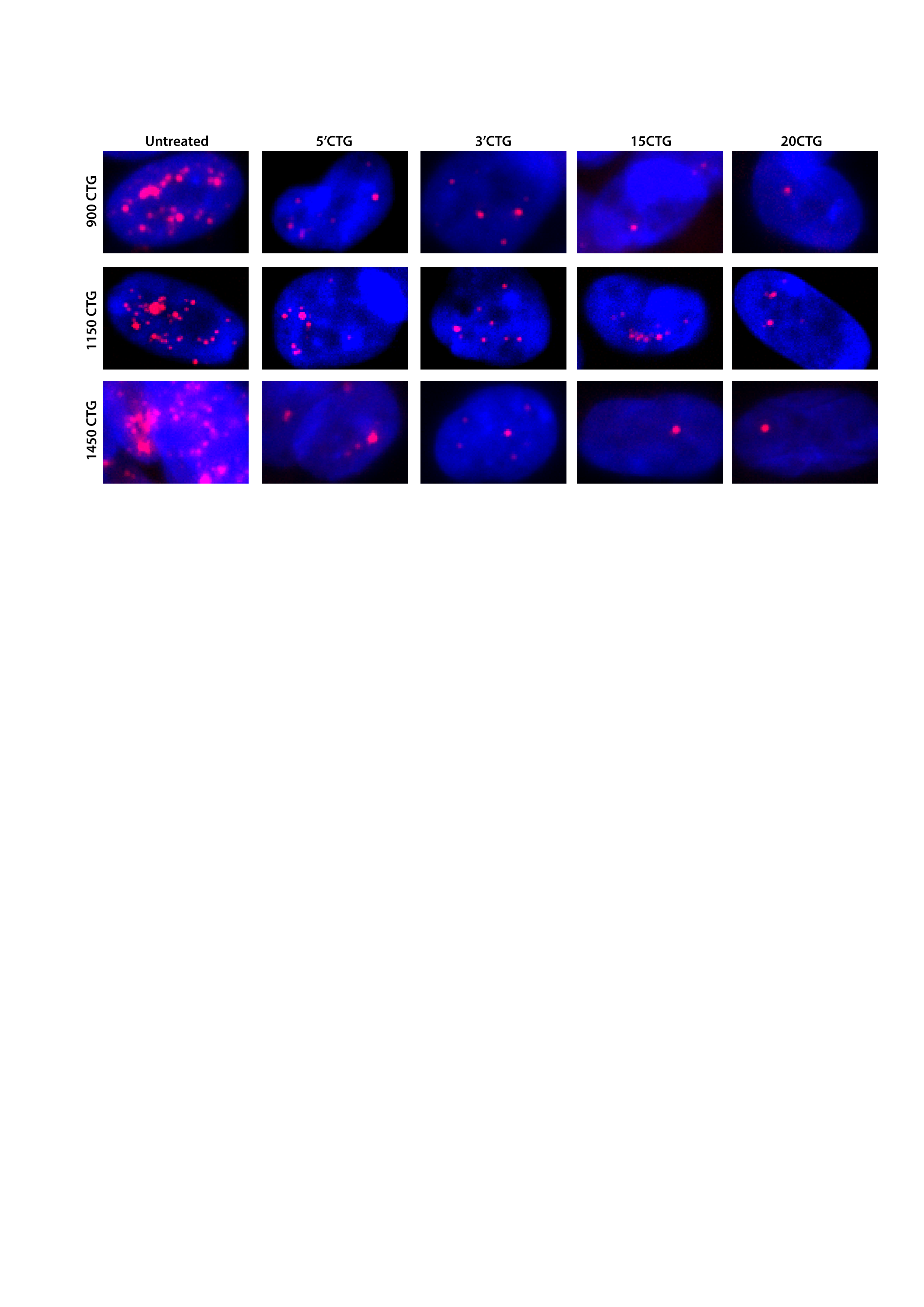

Supplement: Supplementary file 5 [file Image4.TIF]

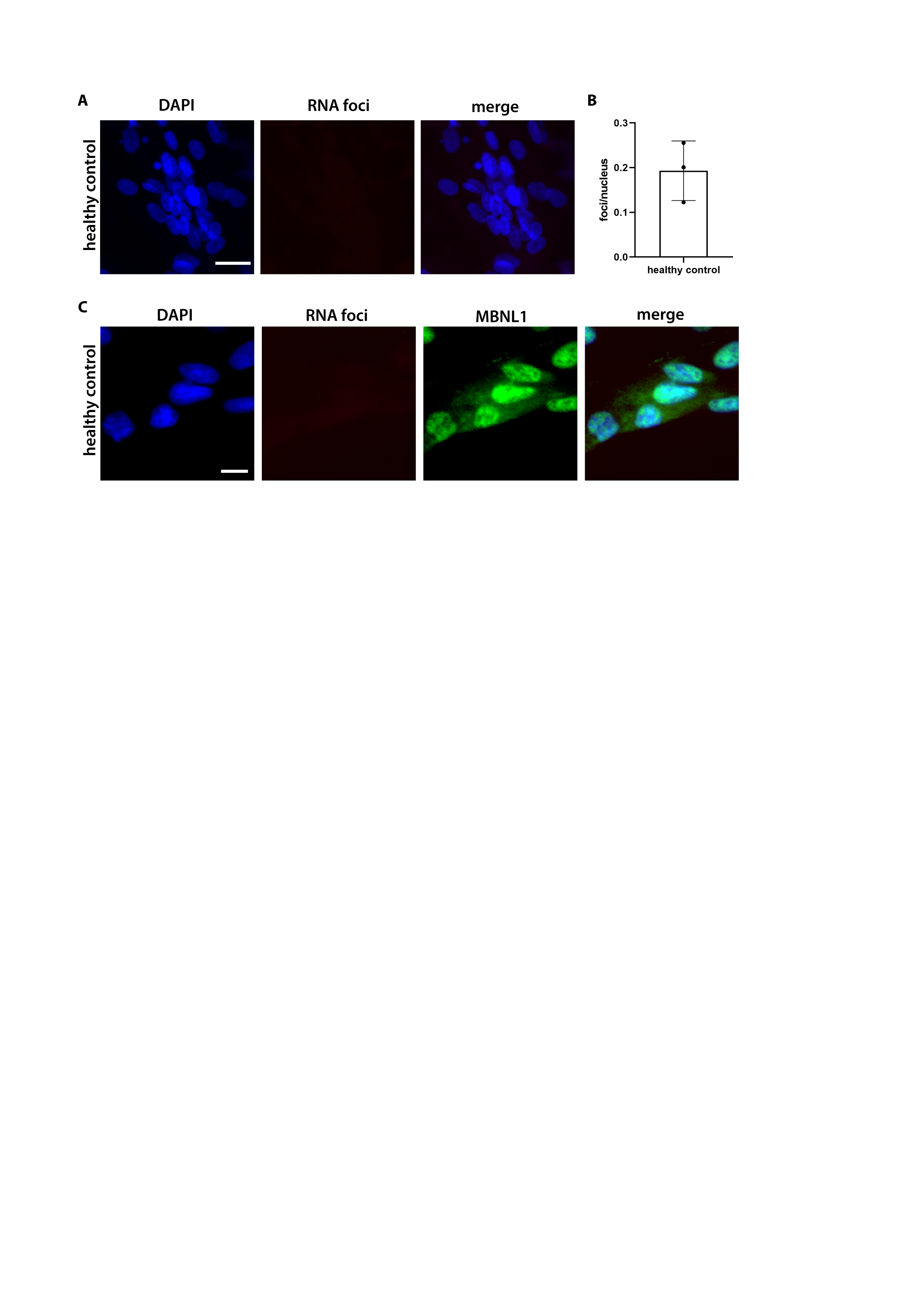

Supplement: Supplementary file 6 [file Image2.TIF]

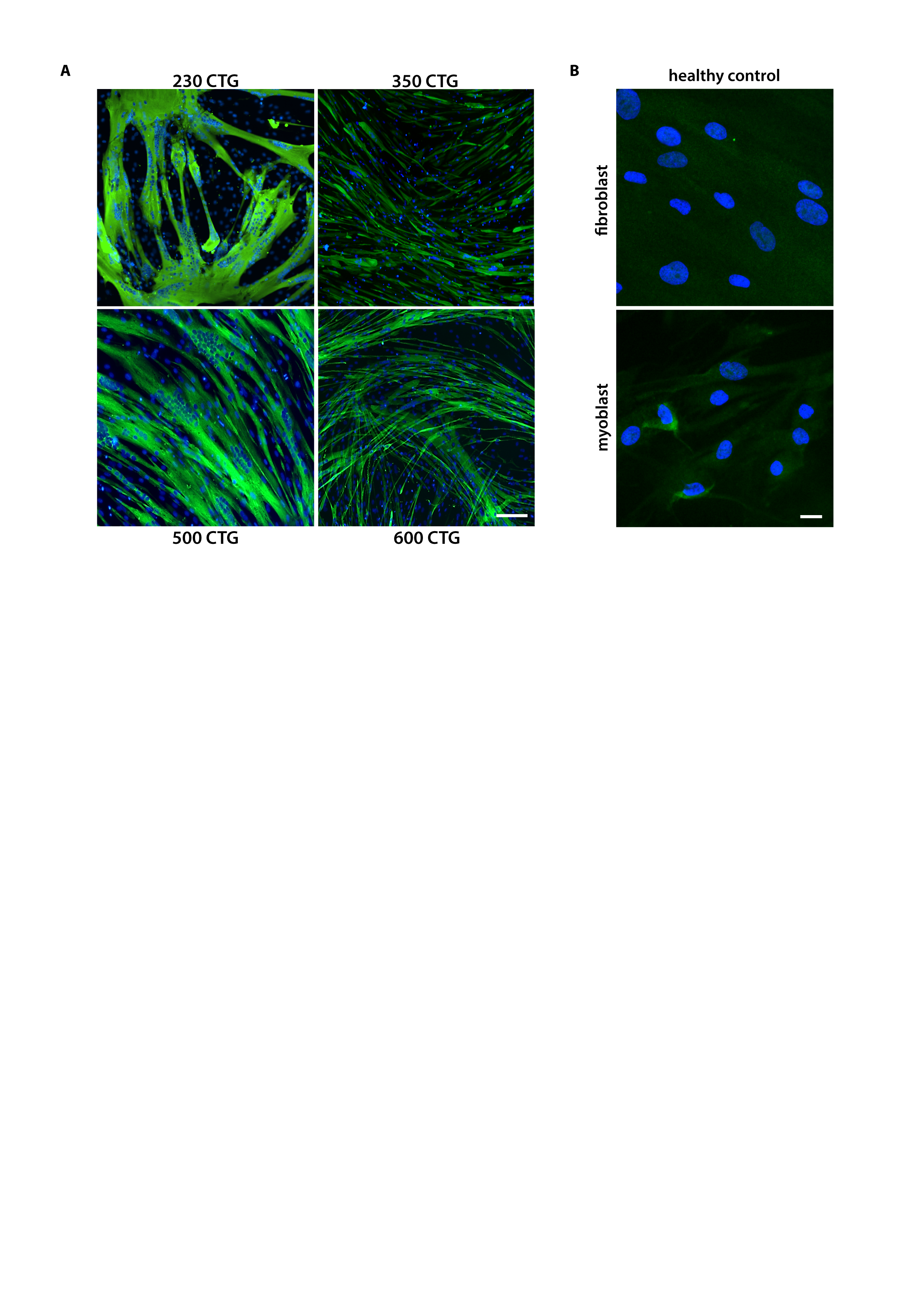

Supplement: Supplementary file 7 [file Image1.TIF]

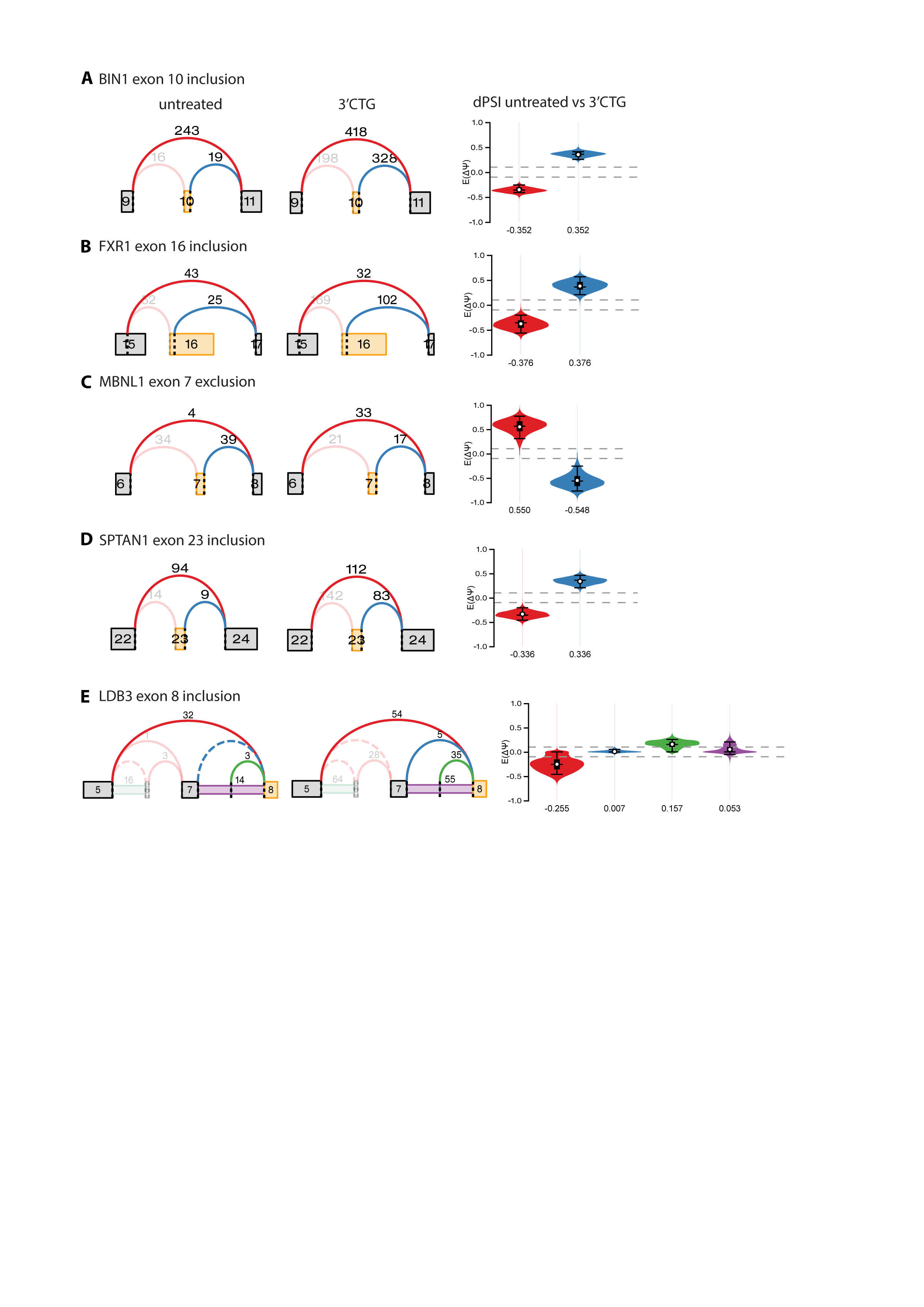

Supplement: Supplementary file 8 [file Image7.TIF]

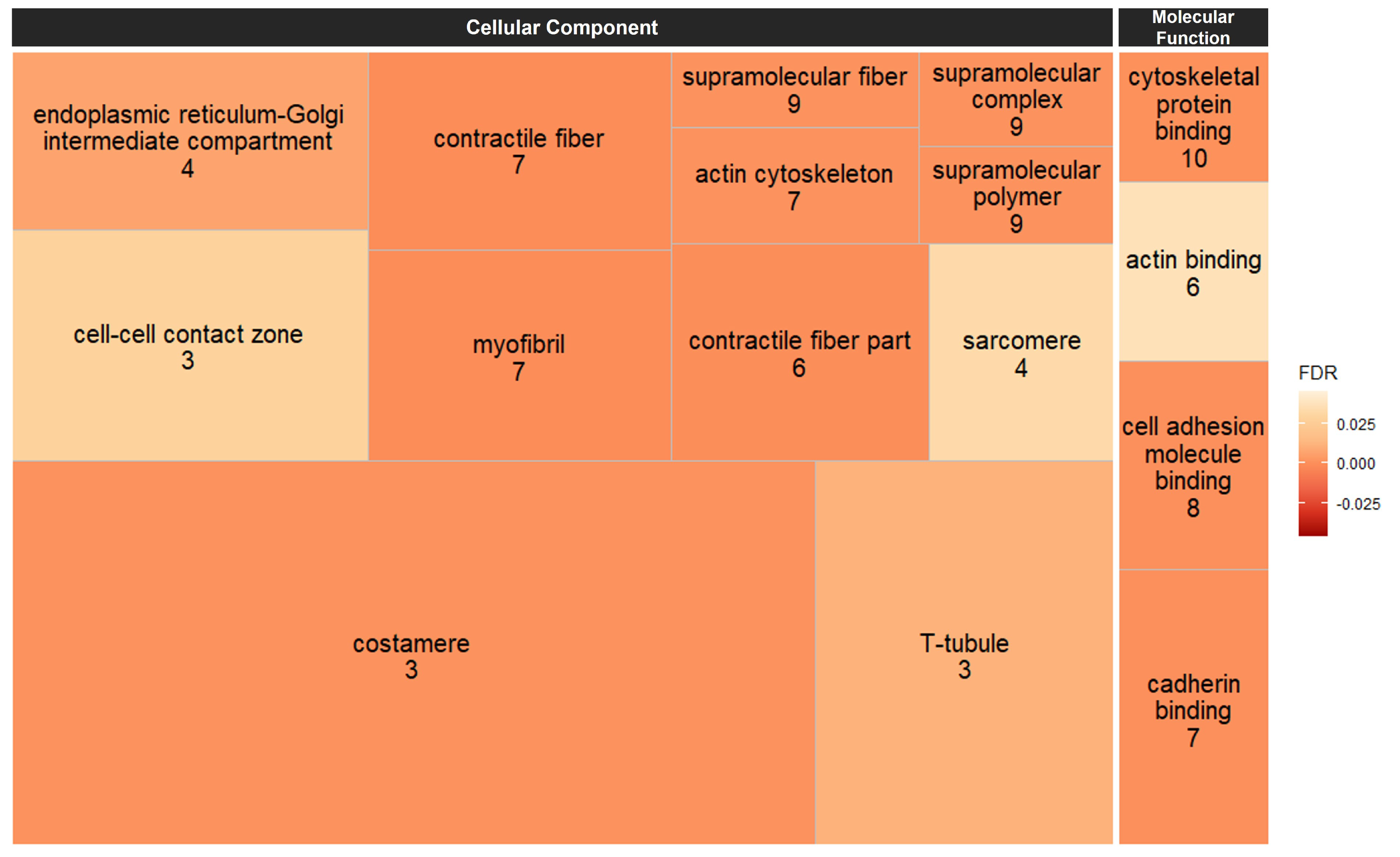

Supplement: Supplementary file 10 [file Image8.TIF]

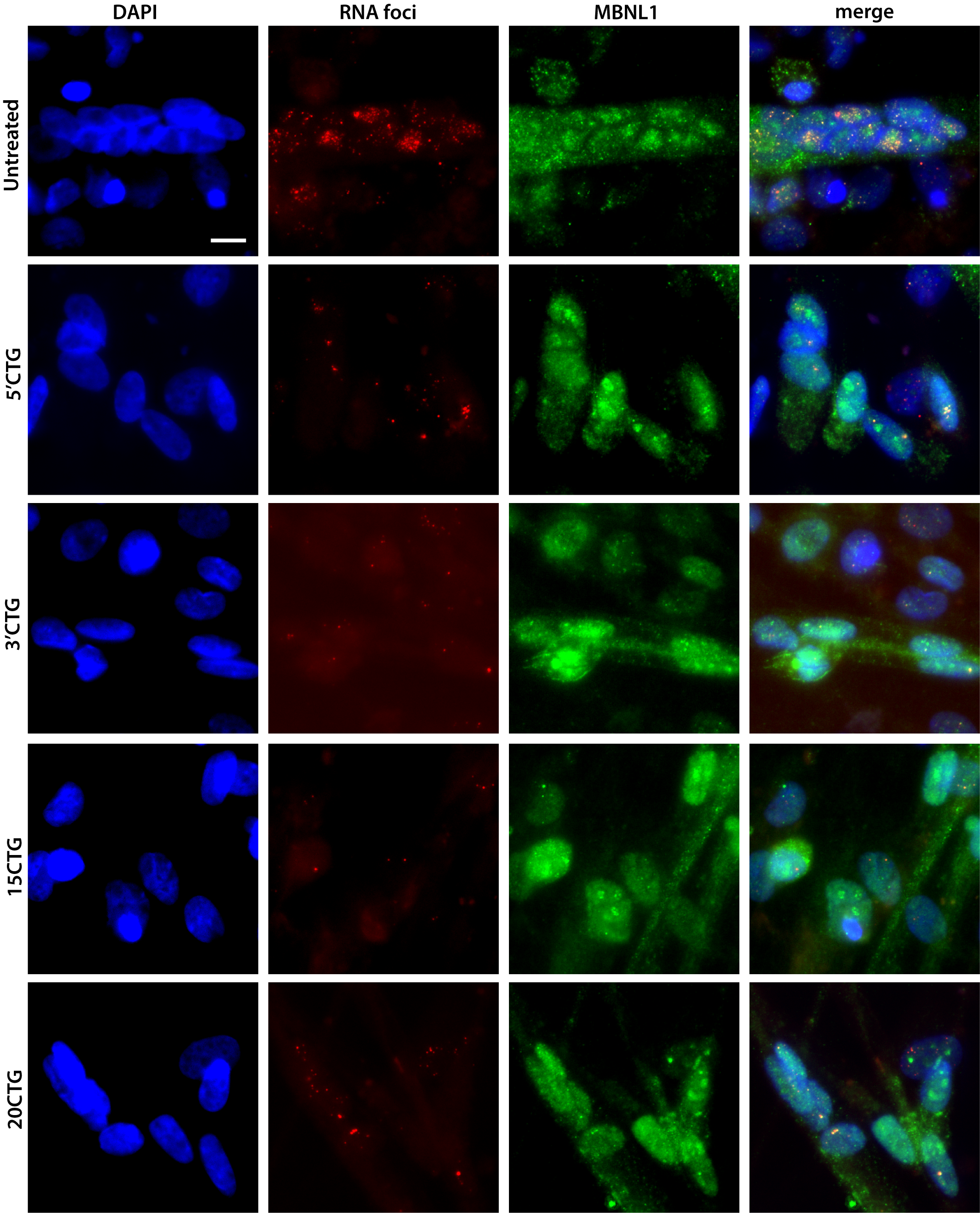

Supplement: Supplementary file 11 [file Image5.TIF]
