# Supplementary material for: Promising AAV.U7snRNAs vectors targeting DMPK improve DM1 hallmarks in patient-derived cell lines
Source: Front Cell Dev Biol. 2023 Jun 15;11:1181040. doi: 10.3389/fcell.2023.1181040 (PMC10309041; doi:10.3389/fcell.2023.1181040)
Supplement: Supplementary file 3 [file Table1.DOCX]

**Supplemental Table 1** Primers sequences for alternative splicing analysis

| Genes | Forward sequence 5' - 3' | Reverse sequence 5' - 3' |
| --- | --- | --- |
| *BIN1* | AGAACCTCAATGATGTGCTGG | TCGTGGTTGACTCTGATCTCGG |
| *DMD* | TTAGAGGAGGTGATGGAGCA | GATACTAAGGACTCCATCGC |
| *IR* | CCAAAGACAGACTCTCAGAT | AACATCGCCAAGGGACCTGC |
| *LDB3* | GCAAGACCCTGATGAAGAAGCTC | GACAGAAGGCCGGATGCTG |
| *MBNL1* | GCTGCCCAATACCAGGTCAAC | TGGTGGGAGAAATGCTGTATGC |
| *MBNL2* | ACAAGTGACAACACCGTAACC | TTTGGTAAAGGATGAAGAGCACC |
| *TNNT2* | ATAGAAGAGGTGGTGGAAGAGTAC | GTCTCAGCCTCTGCTTCAGCATCC |
