# Supplementary material for: Promising AAV.U7snRNAs vectors targeting DMPK improve DM1 hallmarks in patient-derived cell lines
Source: Front Cell Dev Biol. 2023 Jun 15;11:1181040. doi: 10.3389/fcell.2023.1181040 (PMC10309041; doi:10.3389/fcell.2023.1181040)
Supplement: Supplementary file 9 [file Table2.DOCX]

| Cell line |  | Number of CTG repeats |
| --- | --- | --- |
| FM 1 |  | 0 – 37 |
| FM 2 |  | 230 |
| FM 3 |  | 350 |
| FM 4 |  | 500 |
| FM 5 |  | 600 |
| FM 6 |  | 900 |
| FM 7 |  | 1150 |
| FM 8 |  | 1450 |

**Table 2** FibroMyoD cell lines derived from DM1 patients
